# Nitrate acts at the *Arabidopsis thaliana* shoot apical meristem to regulate flowering time

**DOI:** 10.1111/nph.15812

**Published:** 2019-04-17

**Authors:** Justyna Jadwiga Olas, Judith Van Dingenen, Christin Abel, Magdalena Anna Działo, Regina Feil, Anne Krapp, Armin Schlereth, Vanessa Wahl

**Affiliations:** ^1^ Department of Metabolic Networks Max Planck Institute of Molecular Plant Physiology Am Mühlenberg 1 14476 Potsdam Germany; ^2^ Institut Jean‐Pierre Bourgin INRA AgroParisTech CNRS Université Paris‐Saclay 78000 Versailles France

**Keywords:** flowering time, NIN‐LIKE PROTEINs (NLPs), nitrate, nitrate‐responsive elements (NREs), shoot apical meristem (SAM), SQUAMOSA PROMOTER BINDING PROTEIN‐LIKE (SPL), SUPPRESSOR OF OVEREXPRESSION OF CONSTANS (SOC1), trehalose 6‐phosphate (T6P)

## Abstract

- Optimal timing of flowering, a major determinant for crop productivity, is controlled by environmental and endogenous cues. Nutrients are known to modify flowering time; however, our understanding of how nutrients interact with the known pathways, especially at the shoot apical meristem (SAM), is still incomplete. Given the negative side‐effects of nitrogen fertilization, it is essential to understand its mode of action for sustainable crop production.
- We investigated how a moderate restriction by nitrate is integrated into the flowering network at the SAM, to which plants can adapt without stress symptoms.
- This condition delays flowering by decreasing expression of *SUPRESSOR OF OVEREXPRESSION OF CONSTANS 1* (*SOC1*) at the SAM. Measurements of nitrate and the responses of nitrate‐responsive genes suggest that nitrate functions as a signal at the SAM. The transcription factors NIN‐LIKE PROTEIN 7 (NLP7) and NLP6, which act as master regulators of nitrate signaling by binding to nitrate‐responsive elements (NREs), are expressed at the SAM and flowering is delayed in single and double mutants. Two upstream regulators of *SOC1* (*SQUAMOSA PROMOTER BINDING PROTEIN‐LIKE3* (*SPL3*) and *SPL5*) contain functional NREs in their promoters.
- Our results point at a tissue‐specific, nitrate‐mediated flowering time control in *Arabidopsis thaliana*.

## Introduction

Plants take up nutrients from soil throughout their life cycle, providing the potential to grow in tight coordination with the developmental demand (Krouk *et al*., 2011). This is crucial for the timely transition between developmental phases such as the switch from the vegetative to the reproductive phase (Lin & Tsay, 2017). Nitrogen (N) is an essential macronutrient for plant growth, and high yields of almost all cultivated crops require application of large amounts of fertilizer. Hence, N plays an important role in agriculture to improve yield and increase agronomical productivity. However, although fertilizers have helped to increase yield, they also have negative effects, causing environmental and human health problems as well as decreased biodiversity (Smil, 1999; Good & Beatty, 2011; Shibata *et al*., 2015). A surplus of synthetic fertilizers and manure causes emission of nitrous oxide into the atmosphere and leaching of nitrate into soil patches. Many natural water basins and aquifers around the world are currently steering towards severe nitrate pollution through long and continued application of organic and synthetic fertilizers. The maximum contaminant level for public water supply of 50 mg nitrate l^−1^ determined by the World Health Organization (or 10 mg l^−1^ nitrate‐N, stipulated by the US Environmental Protection Agency) is being well exceeded (Ward *et al*., 2018). Reducing the application of fertilizers is a general aim of modern agriculture.

Nitrate is the major source of N for plants and, once taken up by the roots, is distributed within plants by a large number of nitrate transporters. Besides being a nutrient, nitrate itself acts as a signal regulating directly the expression of hundreds of genes (reviewed in Noguero & Lacombe, 2016). These genes encode proteins required for nitrate transport and assimilation, and the reprogramming of carbon (C) and N metabolism, as well as transcription factors and regulatory proteins, triggering a cascade of changes that support increased growth. An early event in nitrate responses involves the accumulation of the transcription factor NIN‐LIKE PROTEIN 7 (NLP7) and its close homolog, NLP6, in the nucleus (Marchive *et al*., 2013; Guan *et al*., 2017), where they bind to nitrate‐responsive elements (NREs) in promoter regions of target genes (Konishi & Yanagisawa, 2013) and regulate their expression.

Yield depends not only on vegetative growth, but also on the optimal timing of reproductive growth. The latter starts with the transition of the shoot apical meristem (SAM) from a vegetative into an inflorescence meristem. This developmental switch is regulated by a complex hierarchical signaling network that integrates many environmental and endogenous stimuli (Blumel *et al*., 2015). Within this network, core pathways have been described that orchestrate responses to day length and light quality (photoperiod pathway), fluctuations in temperature (ambient temperature pathway), exposure to longer periods of cold (vernalization), gibberellic acid signaling (GA pathway), endogenous regulators independent of light and GA (autonomous pathway), and the plant's age (age pathway). Although these signaling pathways have been extensively studied, knowledge of how metabolic signals regulate flowering lags behind. A notable exception is the dependence of the induction of flowering on a plant's energy status. The trehalose 6‐phosphate (T6P) pathway has been shown to convey information about the sucrose status to the flowering network at two signal perception sites. In the leaves, it is necessary and sufficient to induce *FLOWERING LOCUS T* (*FT*), also known as the florigen and a member of the photoperiod pathway, at the end of long days. At the SAM, the T6P pathway interacts with the age pathway both via miR156 and independently of it (Wahl *et al*., 2013).

The nitrate supply is known to modify several developmental processes, including flowering time (Rideout *et al*., 1992; Corbesier *et al*., 2002; Castro Marin *et al*., 2011; Liu *et al*., 2013). The first evidence suggesting that nitrate might be involved in the regulation of flowering time in *Arabidopsis thaliana* was obtained from genetic studies showing that nitrate assimilation and signaling/uptake mutants are late‐flowering (Tocquin *et al*., 2003; Seligman *et al*., 2008). However, interpretation of these observations is challenging as these mutants display severe global metabolic changes, including sugar content, all of which can affect flowering time (Scheible *et al*., 1997; Klein *et al*., 2000). Indeed, studies conducted with *A. thaliana* under various conditions reveal that nitrate has contrasting effects on flowering time depending on the growth system (agar‐, vermiculite‐, soil‐based) and the source of nitrate used (KNO_3_, NH_4_NO_3_, mixed, supplemented with glutamine (Castro Marin *et al*., 2011; Kant *et al*., 2011; Liu *et al*., 2013; Yuan *et al*., 2016; Lin & Tsay, 2017; Gras *et al*., 2018)). Hence, depending on the study, conflicting results were obtained regarding which flowering genes show differential expression in different N regimes, or which flowering pathway mutants alter the response to N, whereas various flowering genes have been reported as correlating with the growth system used but not necessarily responding to nitrate directly (Lin & Tsay, 2017). In a review on the current status of research regarding N‐dependent control of flowering, Lin & Tsay (2017) compiled data published by early 2017 and proposed a U‐shaped response of flowering to nitrate, with an optimal nitrate concentration range for flowering, which is delayed by higher and suboptimal nitrate concentrations (Lin & Tsay, 2017). In addition, flowering is promoted by nitrate starvation (Lin & Tsay, 2017), with the latter being an extreme condition revealing a plant's escape strategy when exposed to stress. Acute stress can either induce or delay flowering (Kazan & Lyons, 2016; Takeno, 2016), defining an emergency exit to secure next generations, should the environmental conditions be too harsh to allow adaptation. A recent review (Takeno, 2016) suggests a separate pathway for stress‐induced flowering, which responds to diverse unsuitable conditions (e.g. poor nutrition, UV light exposure or drought). All these flower‐inducing ‘stresses’ have in common the fact that their corresponding signals, as different as they might be, converge with the flowering network in leaves at the level of *FT*. Taking this into account, Castro Marin *et al*. (2011) supplemented their agar growth system with 4 mM glutamine, in order to be able to vary nitrate but avoid acute N starvation.

Optimal nitrate concentrations differ between growth systems and for species. Furthermore, the concentrations are likely to vary between various tissue types. This might explain why various research groups have proposed different perception sites for nitrate in the flowering time network (Castro Marin *et al*., 2011; Liu *et al*., 2013; Gras *et al*., 2018). Additionally, most previous studies were restricted to analyses on whole rosettes or seedlings instead of tissue‐targeted approaches (e.g. in the SAM) and reflect a great variability in the light and temperature regimes used to grow the plants. Previous research provided evidence that nitrate, once taken up by plants, might interfere with the known flowering network in leaves to modify the flowering time, as a set of flowering genes expressed in leaves (e.g. *FT*,* SMZ*,* SNZ*) was affected by the N status (Castro Marin *et al*., 2011; Lin & Tsay, 2017; Gras *et al*., 2018). However, no evidence has been provided on whether nitrate enters and operates at the SAM.

Tschoep *et al*. (2009) established an N‐limited soil system, which involves growth of plants in an ‘optimal’, full‐nutrition (ON) or ‘low’ nitrate (LN) soil. The concentration of the latter was chosen in such a way that plants were able to adapt to the conditions and maintained a reduced but constant growth over several weeks. This was reflected by the metabolic phenotype, with similar amounts of protein to ON‐grown plants (hereafter ON plants). Analyses of other nutrients, which might potentially affect growth and flowering time (e.g. phosphate or sulfate), demonstrated that their content was unaltered. LN‐grown plants (hereafter LN plants) did not show any visible stress symptoms, such as anthocyanin accumulation, and ON‐grown plants flowered at a similar time to wild‐type (Col‐0) plants on standard soil. Hence, this growth system is an ideal one for studying the effects that N availability might have on flowering time in *A. thaliana*.

Here, we report on a study that made use of the growth system established by Tschoep *et al*. (2009). We found that nitrate is present in the SAM and that nitrate‐regulated genes involved in N assimilation respond in the SAM to N availability. Our data indicate that nitrate acts on the expression of components of the age pathway (i.e. *SQUAMOSA PROMOTER BINDING PROTEIN‐LIKE 3 (SPL3)* and *SPL5*) via NRE motifs in their promoters to induce flowering, and that this process involves the NLP transcription factors NLP6 and NLP7 in the SAM, and leads to differential expression of the flowering integrator gene *SOC1*.

## Materials and Methods

### Plant material

The plants used in this study were *A. thaliana* Columbia (Col‐0, CS76778) wild‐type and mutant and transgenic lines such as *co‐10*,* fd‐3*,* soc1‐6, ft‐10*,* tsf‐1*,* ft‐10/tsf‐1*,* 35S::amiRTPS1 35S::MIR156, nlp7‐1* and *nlp7‐1 nlp6‐2* (Michaels & Amasino, 2001; Rosso *et al*., 2003; Abe *et al*., 2005; Michaels *et al*., 2005; Schwab *et al*., 2005; Yoo *et al*., 2005; Laubinger *et al*., 2006; Lee *et al*., 2007; Jang *et al*., 2008, 2009; J. W. Wang *et al*., 2009; Wahl *et al*., 2013). Genotypes were confirmed (Supporting Information Table S1). Cassettes containing synthetic promoters with four copies of the respective NRE (gene synthesis; Eurofins, Ebersberg, Germany) were designed as previously described (Konishi & Yanagisawa, 2010).

### Growth conditions

Plants were grown in growth chambers (Percival Scientific Inc., Perry, IA, USA) at 22°C under long‐day (LD; 16 h : 8 h, light : dark) or short‐day (SD; 8 h : 16 h, light : dark) conditions with 160 μmol m^−2^ s^−1^ light intensity (Philips F17T8/TL841/Alto). A shift from SD to LD conditions was used to induce flowering as previously described (Schmid *et al*., 2003).

A modified soil‐based N‐limited growth system (ON or LN) was used to grow plants (Tschoep *et al*., 2009). Soil mixtures were stored for at least 2 wk at 10°C before use. The ON and LN soils contained *c*. 31.5 and 1.25 mg inorganic N per 6 cm pot (100 ml or 36 g soil per pot), respectively.

### Phenotypic analyses

Flowering time was defined as days to bolting (DTB) and the total number of leaves (TLN) (Table S2). At least 11 genetically identical plants were used to determine flowering time. Student's *t*‐test was used to test the significance of differences. The leaf initiation rate was calculated by dividing TLN by DTB or recording the number of visible leaves (> 2 mm) (Wang *et al*., 2008). Juvenile leaves were defined as leaves without abaxial trichomes (Telfer *et al*., 1997).

### qRT‐PCR

The total RNA was extracted using a modified phenol : chloroform : isoamyl alcohol (25 : 24 : 1) method as previously described (Wan & Wilkins, 1994). cDNA preparation and quantitative reverse transcription polymerase chain reaction (qRT‐PCR) measurements were performed as previously described (Wahl *et al*., 2013). qRT‐PCR analyses were performed on two to four biological replicates with three or four technical replicates using the Power SYBR^®^ Green‐PCR Master Mix (Applied Biosystems/Life Technologies, Waltham, MA, USA). Relative quantification of gene expression was performed using a comparative cycle threshold (CT) method (Livak & Schmittgen, 2001) with a reference gene index (RGI) and presented as previously described (Wahl *et al*., 2013). Primer sequences are listed in Table S1.

### *GUS* detection, RNA *in situ* hybridization and histological staining

For *GUS* reporter gene detection, seedlings were harvested in 90% ice‐cold acetone, and washed with and incubated in staining buffer (10% Triton X‐100, 0.5 M NaPO_4_, 100 mM K‐ferrocyanide, 100 mM ferricyanide, 100 mM X‐Gluc) overnight at 37°C. Seedlings were dehydrated in an ethanol series, fixed with FAA (formaldehyde, ethanol, acetic acid), washed in 70% ethanol before being embedded in wax. For RNA *in situ* hybridization, apices of plants were collected at the end of the day, fixed and embedded using a Leica system (ASP300S, EG1160; Wetzlar, Germany). Sections of 8 μm thickness were prepared using a rotary microtome (RM2265; Leica). Probes were generated from cDNAs as previously described (Wahl *et al*., 2013). For *LFY*,* SOC1*,* SPL3*,* SPL4* and *SPL5*, full open reading frames containing plasmids were provided by Weigel's and Schmid's laboratories (Weigel *et al*., 1992; R. Wang *et al*., 2009). RNA *in situ* hybridizations and toluidine blue staining was carried out as previously described (Wahl *et al*., 2013).

### Search for NREs in core flowering time genes

Nitrate‐responsive elements (tGACcCTTN_x_AAGagtcc) (Konishi & Yanagisawa, 2010) were searched in the sequences of upstream intergenic regions of known flowering genes with a maximum distance of 3000 bp to the ATG codon. A list of putative NREs is presented in Table S3 and all genes included in the analysis are provided in Table S4.

### Metabolite and nitrate reductase activity measurement

For metabolite measurements, plants were harvested at end of the day and metabolites were analyzed as previously described (Scheible *et al*., 1997; Nunes‐Nesi *et al*., 2007). Nitrate was measured in a coupled enzymatic assay (Cross *et al*., 2006). An adapted protocol was used for SAM samples, from which nitrate was extracted with 70% ethanol once (Stitt *et al*., 1989). Total protein was extracted as previously described (Hendriks *et al*., 2003). Enzyme extraction was performed with some modifications of the buffer ingredients (without triton and BSA) (Gibon *et al*., 2004). The extract was also used for the measurement of total proteins, which were assayed with the Bio‐Rad Bradford reagent according to the manufacturer's instruction (Bio‐Rad). For T6P measurement, tissue samples were extracted and measured as previously described (Lunn *et al*., 2006).

## Results

### Flowering is delayed when nitrate supply is suboptimal

**Figure 1:**
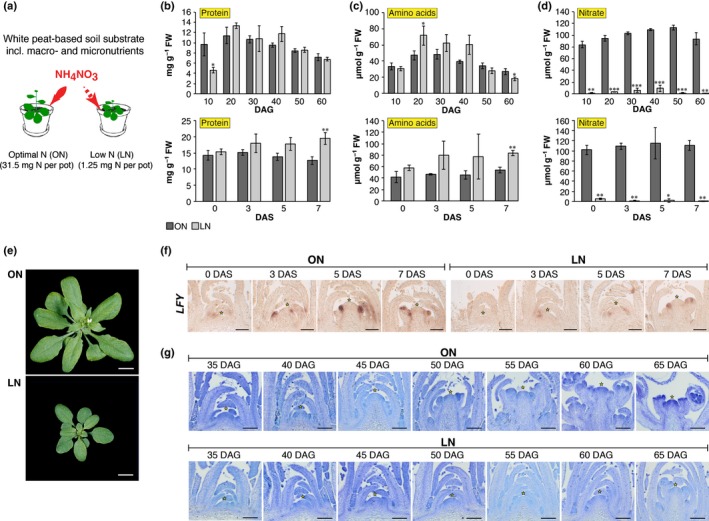
Physiological and morphological analyses of *Arabidopsis thaliana* wild‐type (Col‐0) plants grown in two nitrogen (N) regimes. (a) Simplified schematic model of the previously established, almost natural, soil‐based growth system (Tschoep *et al*., 2009), in which plants are grown in the white peat‐based soil substrate containing a low (1.25 mg per pot, LN) or optimal (31.5 mg per pot, ON) N content. (b–d) Metabolites measured in rosettes of plants grown continuously in short‐day conditions (SD; 8 h light : 16 h dark) or in SD to long‐day (LD; 16 h light : 8 h dark) shift experiments for which plants are grown in SD for 30 d and shifted to LD for 3, 5 and 7 d to induce photoperiod‐dependent flowering. Samples were harvested at the end of the day. Proteins (b) and total amino acids (c) displayed only subtle changes in LN plants. Nitrate concentrations (d) were reduced in LN plants in both sets of experiments. (e) LN plants flowered later than ON plants in all experiments, as demonstrated by flowering time analyses (here for LD grown plants, 25 d after germination (DAG)). (f) Subjecting plants to a SD‐to‐LD shift readily induced *LEAFY* (*LFY*) in ON plants and delayed its expression in LN plants, as demonstrated by RNA *in situ* hybridization. (g) Toluidine blue‐stained longitudinal sections through apices of plants grown in SD conditions demonstrated that floral transition is largely delayed in LN plants compared with ON plants. DAS, days after shift to LD. Error bars indicate SD; statistical significance was calculated using Student's *t*‐test: *, *P* < 0.05; **, *P* < 0.01; ***, *P* < 0.001. Bars: (e) 1 cm; (f, g) 100 μm.

We used a N‐limited soil system (Tschoep *et al*., 2009) (Fig. 1a). ON contains nitrate at a comparable concentration to standard full‐nutrition soil (31.5 mg N per pot) and supports the normal life cycle of wild‐type plants (Tschoep *et al*., 2009). Flowering times for ON‐grown wild‐type plants (Table S2) are comparable with data from studies using standard full‐nutrition soil (± 21 d after germination (DAG) in LD and 61 DAG in SD conditions (Hartmann *et al*., 2000; Lim *et al*., 2004; Kim *et al*., 2007; Wahl *et al*., 2013), demonstrating that the selected nitrate concentration in ON is optimal for plant growth.

**Figure 2:**
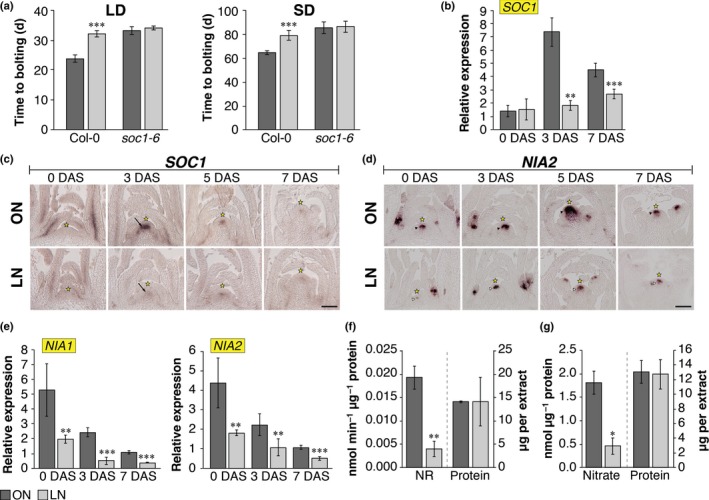
Nitrate regulates flowering time at the shoot apical meristem (SAM) of *Arabidopsis thaliana*. (a) Whereas wild‐type plants are significantly later‐flowering in long‐day (LD) and short‐day (SD) conditions when grown in N‐limited soil (LN), *soc1‐6* plants flower at the same time, indicating that *SUPPRESSOR OF OVEREXPRESSION OF CONSTANS 1* (*SOC1*) is required for the regulation of nitrate‐dependent flowering. (b, c) LN growth causes reduced *SOC1* expression in the SAM of 30‐d‐old SD‐grown plants shifted to LD for 3 and 7 d to induce flowering and harvested at the end of the day, as demonstrated by quantitative real‐time polymerase chain reaction (qRT‐PCR) (b) and by RNA *in situ* hybridization (c) using a specific *SOC1* probe on longitudinal sections through apices (compare arrows in optimal N soil (ON) vs LN at 3 d after shift (DAS)). (d) The strong expression of *NITRATE REDUCTASE 2* (*NIA2*) in the center of the SAM, demonstrated by RNA *in situ* hybridization using a specific *NIA2* probe on longitudinal sections through apices of ON‐grown plants (closed arrow heads in ON), indicates that nitrate assimilation can take place in the SAM. The *NIA2* expression domain is smaller in LN plants (d, open arrow heads in LN). (e) Lower transcript abundance of *NIA1* and *NIA2* was confirmed by qRT‐PCR. (f, g) Nitrate reductase activity (NR) (f) and nitrate (g) measured at the SAM were significantly reduced in LN plants. Both NR and nitrate were calculated on the basis of protein measured in the same extracts, for which no difference was found between the treatments. Error bars denote SD; the statistical significance between ON and LN was calculated using Student's *t*‐test: *, *P* < 0.5; **, *P* < 0.01; ***, *P* < 0.001. Bar, 100 μm.

Plants growing in LN (1.25 mg N per pot) did not display any stress symptoms (e.g. chlorotic leaves or decreased protein and amino acid contents (Figs 1b,c, S1a)), which usually mask direct N‐dependent responses (Tschoep *et al*., 2009). Expression analyses of a stress‐responsive gene by qRT‐PCR did not result in any significant changes between ON and LN plants (Rowan *et al*., 2009) (Fig. S1b). Compared with ON plants, rosettes of LN plants have significantly lower nitrate concentrations (Fig. 1d), are smaller (Fig. 1e) and have a delay in flowering time, with a more profound effect under SD (± 16 d; Fig. 2a; Table S2) than under LD conditions (± 6 d; Fig. 2a; Table S2). When we shifted plants from SD to LD conditions at 30 DAG, a time at which plants reached competence to fully initiate flowering (Schmid *et al*., 2003; Torti *et al*., 2012), LN plants delayed the floral transition by only 2 d as demonstrated by RNA *in situ* hybridization using the floral marker *LEAFY* as a probe (Fig. 1f) and morphologically using stained sections (Fig. S1c). This indicates that LN conditions delay flowering and this effect can be partially overridden by the photoperiod pathway.

The timing of the floral transition at the SAM, as visualized by the increased size of the meristem at transition and the appearance of flower primordia, was comparable to the bolting times we determined for plants grown on either ON or LN with, on average, 16 d difference between the two conditions under SD conditions (Fig. 1g). Similar to previous reports (Tschoep *et al*., 2009; Castro Marin *et al*., 2011; Lin & Tsay, 2017) we found that nitrate supply to plants had an effect on the plastochron length and hence on the production of new leaves, masking the delay when flowering is determined based on TLN instead of DTB (Table S2; Fig. S2). We therefore further determined flowering time based on DTB.

We investigated whether growth in LN also affects the juvenile‐to‐adult phase transition as previously suggested (Vidal *et al*., 2014). This suggestion was based on induction of the expression of some of the miR156 precursors by acute nitrate starvation in whole seedlings (Pant *et al*., 2009; Krapp *et al*., 2011; Liang *et al*., 2012). A delay in the vegetative phase change might explain, at least partially, the late‐flowering phenotype of LN plants. We monitored the vegetative phase change morphologically. The number of juvenile leaves was not altered (Fig. S3a–c), demonstrating that the length of the juvenile phase does not differ between ON and LN plants. However, the levels of two miR156 precursors, *MIR156A* and *MIR156C*, were increased in LN‐grown rosette samples (Fig. S3d). We therefore also analyzed abundance of *SPL* transcripts, which are the targets of miR156 (Rhoades *et al*., 2002). SPLs belong to a large family of transcription factors, several of which play a role in age‐dependent developmental transitions such as the vegetative phase change and the floral induction as part of the age pathway (Xu *et al*., 2016). We did not detect significant changes between ON and LN plants in rosettes, except for increased levels of *SPL4* in LN plants (Fig. S3e). Increased expression of *SPL4* in the rosettes of LN plants is inconsistent, with a delayed vegetative phase change; whereas miR156 levels decline when plants age, *SPL* transcript abundances increase (Wang, 2014). Together, these results show that limiting N in a nonstressful way delays flowering time but not the vegetative phase change.

### SOC1 is required for the regulation of N‐dependent flowering

To determine the potential contribution of known flowering pathways to nitrate‐dependent flowering, we performed flowering time analyses of mutants in components of different pathways of the flowering network and wild‐type plants grown in the two N regimes (Table S2). Interestingly, within our initial set, we identified only one mutant line that behaved differently from Col‐0 wild‐type plants in that its flowering time did not respond to the N treatment. A mutation in the *SOC1* locus caused ON and LN plants to flower at the same time in both LD and SD conditions (Fig. 2a; Table S2). This observation indicates that nitrate‐dependent flowering requires a functional SOC1. Interestingly, flowering data from Castro Marin *et al*. (2011) had already indicated that the *soc1‐1* mutant (L*er* background) grown on agar plates displays a reduced response to nitrate. However, as the wild‐type L*er* accession itself presented only a weak response to nitrate in their conditions, the authors did not conclude from their results that SOC1 contributes to nitrate‐dependent regulation of flowering.

SOC1 serves as a central integrator for multiple flowering pathways in the SAM (Srikanth & Schmid, 2011) and its expression is strongly upregulated in the center of the meristems when plants are shifted from noninductive SD conditions to inductive photoperiods (LD). To further examine the role of SOC1 in nitrate‐dependent flowering, we grew plants in ON and LN under SD conditions for 30 d and then shifted them to LD conditions and analyzed the expression of *SOC1* at the SAM by qRT‐PCR analysis and RNA *in situ* hybridization using *SOC1* as a probe (Fig. 2b,c). *SOC1* expression was upregulated in response to an inductive photoperiod at a much later time point and had greatly reduced levels in LN plants, indicating that SOC1 is an essential player in nitrate‐dependent flowering at the SAM.

These findings contradict with an earlier study carried out using an agar growth system (Liu *et al*., 2013). Here the authors demonstrated that low nitrate concentrations (1 mM nitrate and 4 mM glutamine, agar‐based) induce the expression of *SOC1* in rosette samples at a 10‐leaf stage in a 12 h : 12 h light : dark photoperiod, and suggested that this induction is mediated by an increase in the biosynthesis of GA, causing an early‐flowering phenotype (Liu *et al*., 2013). In addition, another study (1 mM vs 3 mM KNO_3_, vermiculite‐ or agar‐based) suggested that GA signaling is involved in the late‐flowering phenotype caused by high nitrate content (Gras *et al*., 2018). In order to discover whether the GA pathway is affected, five transcripts encoding components of the GA biosynthesis and its signaling were analyzed in SD conditions. We did not detect any differences between ON and LN plants (Fig. S4), demonstrating that nitrate does not interact with GA biosynthesis and signaling when plants have the chance to adapt to available nitrate conditions in soil.

Nitrogen and C are the most important elements to establish normal plant growth and development, and an intricate crosstalk between their signaling networks exists to maintain a balance between N and C metabolism (Nunes‐Nesi *et al*., 2010). C in the form of sucrose is translated to the flowering network and diverse metabolic pathways via the T6P pathway (Wahl *et al*., 2013; Figueroa *et al*., 2016), suggesting that it is a hub within an N‐/C‐sensory checkpoint. The T6P pathway is required for expression of *FT* and affects the age pathway at the level of *SPL3‐5* partially via the miR156 (Wahl *et al*., 2013). Knockdown plants of *TREHALOSE PHOSPHATE SYNTHASE 1* (*TPS1*,* 35S::amiRTPS1*), which encodes the enzyme producing T6P, still responded to LN (Fig. S5a; Table S2). This indicates that nitrate‐dependent flowering acts in parallel with the T6P pathway to regulate flowering time. Further, mutants in *FT*,* TWIN SISTER OF FT* (*TSF*), *CONSTANS* (*CO*), *FLOWERING LOCUS D* (*FD*) and miR156‐overexpressing plants showed delayed flowering in LN compared with ON conditions (Fig. S5b–d; Table S2), demonstrating that the photoperiod and the age pathway are not involved in nitrate‐dependent flowering. LN also had only a marginal effect on transcript abundances of genes assigned to the photoperiod pathway (Fig. S5e, f) including known floral repressors (e.g. *SMZ*), which have previously been suggested to be involved in the late‐flowering phenotype of plants grown in a high‐N content growth regime (Gras *et al*., 2018).

As already mentioned, wild‐type plants show a much weaker response to LN in LD than in SD condtions, where the promoting effect of the photoperiod pathway is absent (Fig. 2a; Table S2). Further evidence that the photoperiod pathway is not involved in and may even partly override the LN response is provided by two further observations. First, *ft/tsf* double mutants, which are blocked in the photoperiod response, showed a late‐flowering phenotype in LN under LD conditions (± 70 d), which resembled ON‐grown wild‐type plants under SD conditions (Fig. S5d; Table S2). Second, ON and LN *ft/tsf* plants under LD conditions showed a similar late‐flowering response to that of wild‐type plants under SD conditions (Figs 2a, S5d).

### Nitrate assimilation at the SAM is regulated by N availability

To date, published data have demonstrated that nitrate after uptake in the roots can be assimilated, stored in vacuoles or transferred to different parts of the plant to foster growth (Tischner, 2001). However, no evidence has come to light on whether nitrate enters and is assimilated in the SAM. If this were to occur, it would open up the possibility that the available nitrate in the SAM can directly regulate flowering genes. In an attempt to understand whether meristematic tissue contains and can assimilate nitrate, we examined the expression of *NITRATE REDUCTASE 1* and *2* (*NIA1* and *NIA2)* genes by RNA *in situ* hybridization and qRT‐PCR in the SAM (Fig. 2d,e). They encode enzymes catalyzing the first step in nitrate assimilation (Krapp *et al*., 2014). *NIA1* and *NIA2* are nitrate‐induced genes and belong to the primary nitrate response, with changes occurring within 30 min. This first wave of the transcriptional response to nitrate does not require *de novo* protein synthesis or the presence of nitrate reductase, showing that these transcripts respond directly to nitrate (Gowri *et al*., 1992). Using transcript‐specific probes for *NIA1* and *NIA2* on longitudinal sections of the apex, we found that both genes are expressed in the SAM (Figs 2d, S6a,b). Transcript of *NIA1* was detected in leaves, the borders of the rib (RZ) and peripheral (PZ) zones of the SAM and the axillary meristem, whereas the transcript of *NIA2* was detected in the center of the SAM, stretching from L3 into the RZ, and no transcript was detected in the PZ. Most importantly, using both RNA *in situ* hybridization and qRT‐PCR, we found that expression of both genes was greatly reduced in LN compared with ON plants (Figs 2d,e, S6a,b), indicating that nitrate is regulating *NIA* expression in the SAM of these plants. This was corroborated by activity measurements in excised apices grown in SD conditions for 30 d (Fig. 2f) and by measurements of nitrate, which revealed significantly reduced concentrations in the SAM of LN vs ON plants (Fig. 2g). With these findings we provide the first evidence that nitrate metabolism can take place in the SAM proper and that nitrate‐dependent signaling might directly interact with processes at the SAM.

### Nitrate‐dependent regulation of *SPL* gene expression at the SAM

**Figure 3:**
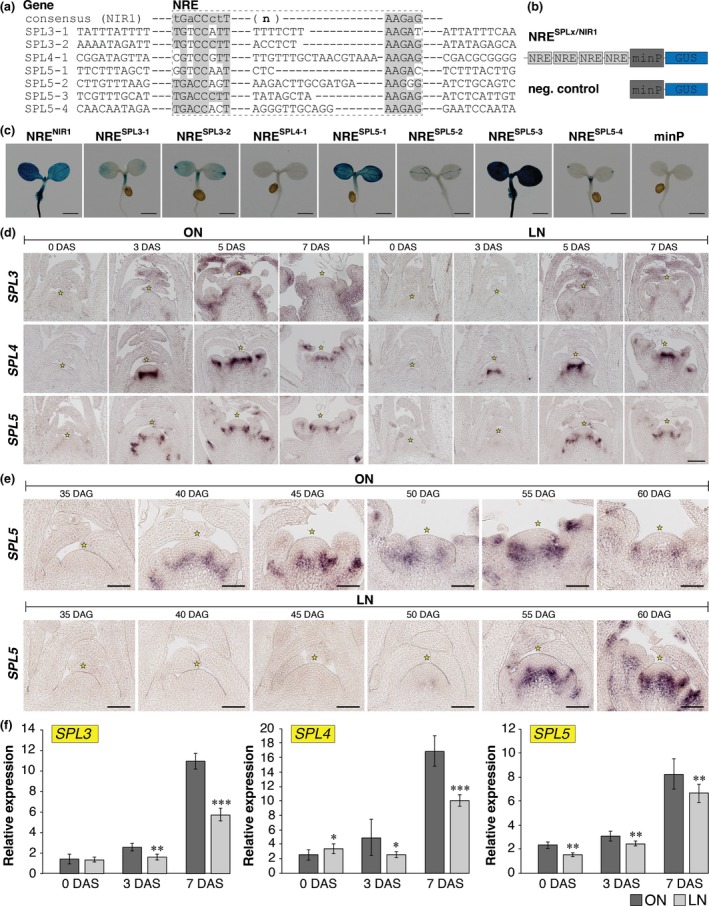
Nitrate‐dependent expression of *SQUAMOSA PROMOTER BINDING‐LIKE* (*SPL*) genes at the shoot apical meristem (SAM) of *Arabidopsis thaliana*. (a) Putative nitrate‐responsive elements (NREs) in the upstream regulatory regions of *SPL3*,*SPL4* and *SPL5*. (b) Schematic illustration of expression cassettes consisting of four copies of the respective NREs fused to a 35S minimal promoter (minP) driving a *GUS* reporter gene and negative control without NRE. (c) Histological staining of the synthetic promoter‐*GUS* lines compared with the negative control. (d) RNA *in situ* hybridization using specific probes for *SPL3*,*SPL4* and *SPL5* on longitudinal sections through apices of plants grown in the two nitrogen (N) regimes (optimal N soil (ON) and limited N soil (LN)) in short‐day (SD) conditions for 30 d, before shifting them to long days (LD) for 3, 5 and 7 d (DAS, days after the shift). Bar, 100 μm. (e) RNA *in situ* hybridization using a specific probe for *SPL5* on longitudinal sections through apices of plants grown in continuous SD conditions and harvested at the end of the day. (f) Transcript abundances of *SPL3*,*SPL4* and *SPL5* measured by quantitative reverse transcription polymerase change reaction in apices. Bars: (c) 1 mm; (d) 100 μm; (e) 50 μm. Error bars denote SD; the statistical significance between ON and LN was calculated using Student's *t*‐test: *, *P* < 0.05; **, *P* < 0.01; ***, *P* < 0.001.

Sequence analysis in several plant species has identified a conserved motif, termed the nitrate‐responsive *cis*‐element (NRE), in the upstream regions of many nitrate‐induced genes (Konishi & Yanagisawa, 2010). The NRE sequence has been reported to be both necessary and sufficient for nitrate induction (Konishi & Yanagisawa, 2011). In addition, Konishi & Yanagisawa (2011) found that NRE‐dependent transcription is only activated by nitrate and not by other N sources. We analyzed upstream intergenic regions of core players of the flowering network for the existence of putative NRE motifs (Table S4) and identified such motifs predominantly in *SPL3*,* SPL4* and *SPL5* (Fig. 3a; Table S3), which are expressed at the SAM during the floral transition (Schmid *et al*., 2003). NRE motifs were not found in the upstream intergenic regions of other *SPL*s, such as *SPL9* or *SPL15*, which are also described to control flowering (Hyun *et al*., 2016). We generated transgenic plants harboring synthetic promoters with four copies of the respective NRE motif fused to a 35S minimal promoter driving the *GUS* reporter gene (Konishi & Yanagisawa, 2010) (Fig. 3b). Histochemical staining of seedlings grown for 4 d on full nutrition ½ Murashige & Skoog medium (10.3 mM NH_4_NO_3_, 9.4 mM KNO_3_) resulted in strong stains for the positive control (NRE^NIR1^) as well as for NRE^SPL5‐1^ and NRE^SPL5‐3^, weaker stains for NRE^SPL3‐1^, NRE^SPL3‐2^, NRE^SPL5‐2^ and NRE^SPL5‐4^, and no stain for NRE^SPL4‐1^ and the negative control (Fig. 3c). These results demonstrate that the NRE motifs present in the upstream intergenic regions of *SPL3* and *SPL5* can activate transcription.

We next analyzed *SPL* expression via RNA *in situ* hybridization in the SAM after transferring ON or LN plants from SD to inductive LD conditions. Compared with ON plants, LN plants showed a delay in the rise of *SPL3* and *SPL5,* but not *SPL4* transcript (Fig. 3d). Also, under continuous SD conditions the rise in expression of *SPL5* at the SAM was delayed in LN vs ON plants (Fig. 3e). qRT‐PCR analyses on apices confirmed this result (Fig. 3f), indicating that timely expression of *SPL3* and *SPL5* at the SAM requires a positive input by nitrate signaling. In addition, *SPL4* expression was reduced at the SAM of LN plants (Fig. 3f), suggesting a tissue‐specific, but presumably NRE‐independent, effect of N supply on the regulation of *SPL4*.

**Figure 4:**
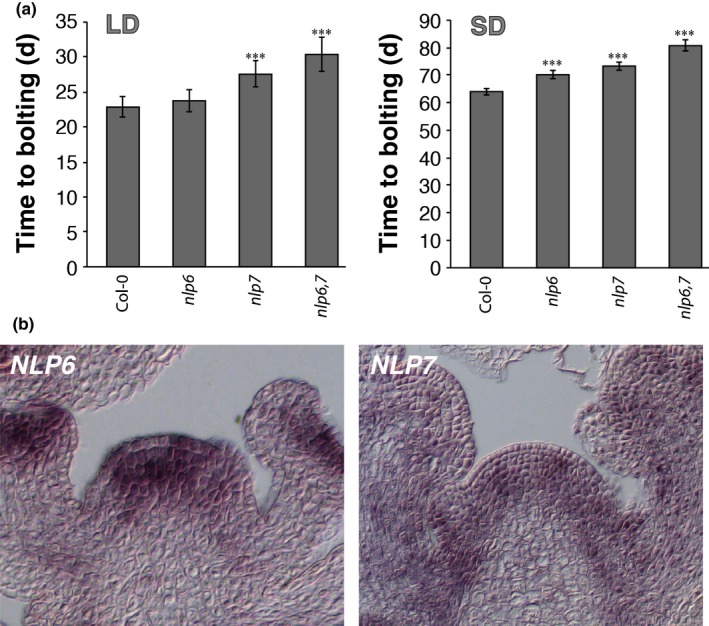
Master regulators of nitrate signaling are present at the shoot apical meristem (SAM) of *Arabidopsis thaliana*. (a) Flowering time analyses of plants mutant for *NIN‐LIKE PROTEIN 6* (*NLP6*) and *NLP7* based on ‘time to bolting’ (d), determined in long‐day (LD) and short‐day (SD) conditions on standard full‐nutrition soil. (b) RNA *in situ* hybridization on longitudinal sections through inflorescence apices of plants grown in LD conditions using specific probes against *NLP6* and *NLP7*. Error bars denote SD; the statistical significance between optimal N soil (ON) and limited N soil (LN) was calculated using Student's *t*‐test: ***, *P* < 0.001.

NIN‐LIKE PROTEIN transcription factors bind to NREs in the presence of nitrate via their RWP‐RK DNA‐binding domain to affect expression of downstream nitrate‐responsive genes (Krapp *et al*., 2014). NLP7 is known as the master regulator of nitrate signaling and is controlled at a subcellular level by a nitrate‐dependent nuclear retention mechanism (Marchive *et al*., 2013). NLP6 function is described as partially redundant to NLP7 (Guan *et al*., 2017). A late‐flowering phenotype has been observed for the *nlp7* mutants (Castaings *et al*., 2009). When we grew *nlp6* and *nlp7* mutant plant lines on full‐nutrition soil in LD and SD conditions, we found that, for both mutants, flowering time was delayed compared with wild‐type plants, whereas the double *nlp6 nlp7* mutant flowered significantly later than either of the single mutant lines (Fig. 4a; Table S2). Furthermore, using RNA *in situ* hybridization, we detected *NLP6* and *NLP7* expression at the SAM (Fig. 4b). These results indicate that both NLPs are candidates for the direct regulation of *SPL3* and *SPL5* via the NREs in their promoters, suggesting a novel mechanism by which the nitrate content in the SAM acts directly to regulate expression of flowering time genes and promote flowering.

### Flowering time in LN plants under SD conditions depends on the T6P pathway

**Figure 5:**
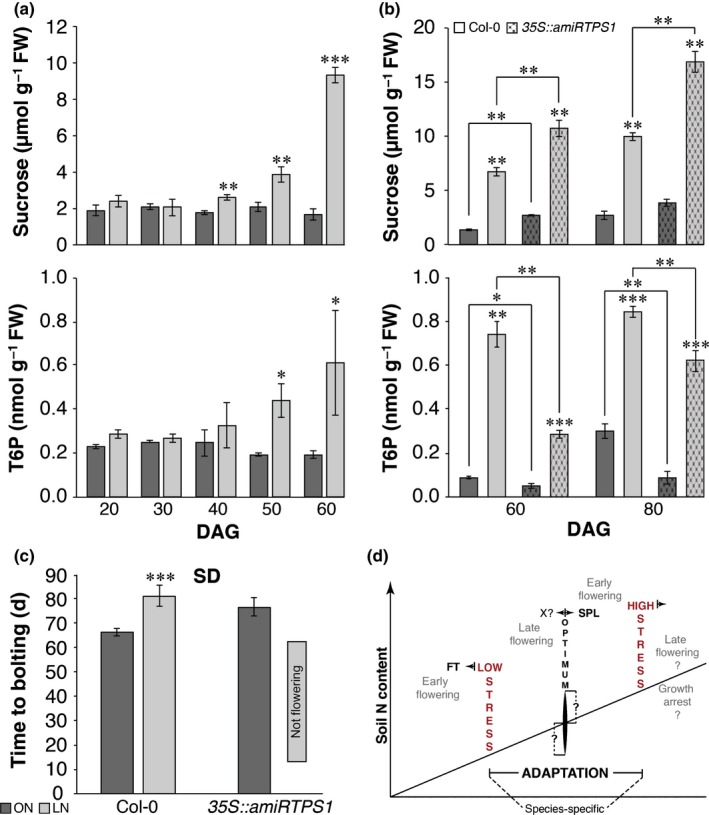
Nitrate‐signaling and the trehalose 6‐phosphate (T6P) pathway act independently to control flowering in *Arabidopsis thaliana*. (a–c) Sucrose and T6P concentrations were measured in rosettes of wild‐type plants grown in the two nitrogen (N) regimes in short‐day conditions (SD) and harvested at the end of the day (a, b). Sucrose and T6P (a) concentrations rose in plants grown in limited N soil (LN plants), whereas the concentrations stayed constant in plants grown in optimal N soil (ON plants) throughout the experiment and only appeared to be significantly changed in wild‐type plants in very old, senescing plant material (compare dark gray columns and 60 vs 80 d after germination (DAG) in panel b). As previously reported, sucrose and T6P concentrations are higher and lower (b), respectively, in rosettes of nonflowering *35S::amiRTPS1* line grown in LN (c) (Wahl *et al*., 2013). (d) Hypothetical graph on the species‐specific relationship between N abundance and its effect on plant growth and flowering time in plants. Error bars denote SD; the statistical significance was calculated using Student's *t*‐test: *, *P* < 0.05; **, *P* < 0.01; ***, *P* < 0.001.

As mentioned before, in LD conditions the photoperiod pathway may partly override the negative effect of LN and lead to flowering, albeit with a delay. LN plants also eventually initiated flowering in SD conditions, when the photoperiod pathway was inactive (Fig. 5c). This implies that in SD conditions a further flower‐inducing signal overrides low N. We noticed that sucrose and T6P concentrations increased in rosettes of LN wild‐type plants grown under SD conditions towards the end of the growth phase (Fig. 5a). This finding is consistent with an earlier study showing that nitrate starvation affects T6P concentrations in a liquid culture with seedlings (Yadav *et al*., 2014). To test whether this rise in T6P overrides the effect of low N, we grew *35S::amiRTPS1* plants in LN under SD conditions. T6P concentrations increased in LN‐grown *35S::amiRTPS1* plants (Fig. 5b) but the rise was delayed and T6P concentrations remained below wild‐type concentrations. Strikingly, the *35S::amiRTPS1* plants never flowered (Fig. 5c; Table S2). We previously reported that constitutive expression of *MIR156b* in the background of *35S::amiRTPS1* plants yields plants that are unable to flower on standard soil in SD conditions (Wahl *et al*., 2013). Growth in LN leads to decreased expression of the *SPL*s in the SAM by limiting nitrate (Fig. 3d–f). It is likely that the nonflowering phenotype of LN‐grown *35S::amiRTPS1* plants in SD conditions is due to a stalled T6P pathway in combination with decreased *SPL* expression. Taken together, our data demonstrate that floral induction in the late‐flowering wild‐type plants grown under nitrate‐limited and SD conditions largely relies on the T6P pathway. If both pathways are impeded, flowering cannot occur, highlighting the joint importance of the nitrate signaling pathway and the T6P pathway for the onset of flowering.

## Discussion

Understanding the molecular mechanisms underlying the regulation of nitrate‐dependent flowering is a crucial step towards the development of alternative breeding strategies for a sustainable production of staple crops under nitrate‐limited conditions. In contrast to the growth systems used in previous studies (reviewed in Lin & Tsay, 2017), we used a soil‐based low‐N system (Tschoep *et al*., 2009) in which plants are able to adapt their metabolism and growth to the reduced N supply. This allowed us to study the control of flowering time without complications due to stress or major changes in central metabolite concentrations. LN prolongs the adult vegetative phase and delays flowering of *A. thaliana*, but the length of the juvenile vegetative phase is not altered. As seen previously, expression of some of the miR156 precursors are increased in LN leaves (Pant *et al*., 2009; Krapp *et al*., 2011; Liang *et al*., 2012), although expression levels of miR156 targets do not change in response to LN, contradicting earlier assumptions (Vidal *et al*., 2014). However, we cannot exclude the possibility that these changes become relevant at more extreme nitrate conditions, as predicted by Lin & Tsay (2017).

We found that none of the flowering pathways originating in leaves caused the delayed flowering phenotype in LN. This includes all members of the photoperiod pathway as well as its repressors, although these have recently been associated with high N‐dependent flowering (Gras *et al*., 2018). The SAM produces all of the aerial organs of a plant and, as such, major changes related to the floral transition, that is the production of flowers instead of leaves, are realized at the SAM. However, some of the signals that control this transition are initiated in leaves (Srikanth & Schmid, 2011). Indications that nitrate directly acts as a signal for flowering were already found in previous studies in which supplementation of a limited nitrate medium with glutamine did not rescue the flowering phenotype (Castro Marin *et al*., 2011; Weber & Burow, 2018). However, evidence that nitrate signaling can directly interact with the flowering network at the SAM has not yet been reported. A recent publication describes a root‐borne cytokinin signal that transduces nitrate availability to the SAM within a matter of days and controls the stem cell population and hence meristem size and growth (Landrein *et al*., 2018). It remains an open question whether this systemic signal also contributes to the regulation of flowering as previously suggested (D'Aloia *et al*., 2011). Our findings demonstrate that nitrate does not necessarily need a second messenger to transduce its status to the flowering network in the SAM. The local sensing of nitrate at the SAM, as strongly suggested by our study, will allow changes in the meristematic nitrate concentration to be rapidly translated into downstream events, allowing high developmental plasticity in fluctuating environmental conditions.

We found that nitrate reaches the SAM, where it can be assimilated, as also demonstrated by the presence of *NIA1* and *NIA2* transcripts and nitrate reductase activity measured in excised apices. These genes, which are part of the primary transcriptional response to nitrate, are repressed in the SAM under limited nitrate availability. We also found that NLP transcription factors, which convey nitrate content information to important downstream targets within the flowering network, are present in the SAM. Both *SPL3* and *SPL5* carry two and five functional NRE sequences in their upstream intergenic regions, respectively. These elements are targeted by NLP6 and NLP7 upon activation and nuclear retention in the presence of nitrate (Marchive *et al*., 2013; Guan *et al*., 2017). NLP6 and NLP7 belong to a family of nine nitrate‐regulated transcription factors present in the *A. thaliana* genome, and all nine members can potentially bind to NREs (Konishi & Yanagisawa, 2013). Currently, NLP7 is considered the master regulator for nitrate signaling, conveying information of a cell's nitrate status to metabolic and developmental processes (Castaings *et al*., 2009). However, other NLPs have also been described to function in distinct developmental processes (Yu *et al*., 2016). Given that they are expressed in almost all plant organs (Winter *et al*., 2007; Castaings *et al*., 2009; Chardin *et al*., 2014), it will be interesting to uncover the functional significance of each of the NLPs in the future. Although we did not identify a functional NRE in *SPL4* in the seedling GUS assay, its expression is decreased at the inflorescence SAM of LN plants, suggesting a different signaling mechanism. For the time being we also cannot exclude an additional contribution of miR156, as at least *MIR156A* and *MIR156C* levels were slightly but significantly increased in leaves of LN plants at some times in our analyses. Interestingly, none of the observed changes in leaves or the SAM led to a prolongation of the juvenile vegetative phase.

Two lines of evidence support the idea that a functional SOC1, a central integrator for several flowering pathways at the SAM, is required for the nitrate response; first, *soc1‐6* mutant plants flower at the same time in both growth regimes and, second, *SOC1* expression is severely reduced in wild‐type plants under nitrate‐limited conditions. *SOC1* transcript increases in *35S::MIM156* plants, arguing for an effect of the age pathway on *SOC1* expression at the SAM (J. W. Wang *et al*., 2009). In addition, the *SOC1* genomic region contains several GATC boxes, which are known target sequences of SPLs, and overexpression of a miR156‐resistant version of *SPL3* led to an increased expression of *SOC1*. We therefore postulate an indirect nitrate effect on *SOC1* via transcriptional regulation of *SPL3* and *SPL5* through NLP6 and NLP7. Interestingly, SOC1 was also shown to feed back on *SPL3‐5* independently of miR156 by directly interacting with their CArG boxes (Jung *et al*., 2016).

In addition to the NRE motifs present in the upstream intergenic regions of *SPL3* and *SPL5*, we found more potentially interesting NRE motifs in 5′ intergenic regions of other flowering time genes (e.g. *SMZ*,* TOE2*,* TOE3* and *MIR156F*). However, no significant or relevant changes in expression of those genes were observed in the conditions used in our study, which is why we did not pursue them further for functional relevance. The plants grown on limited N in our study did not suffer from acute nitrate starvation. This may explain why we did not find some of the targets reported in studies that used more severe conditions. We assume that changes of some of these genes, in particular regulators of *FT*, become relevant only when N supply is more strongly reduced and triggers a general stress level, that is, conditions in which flowering is reported to be induced rather than delayed relative to ON. For example, in a recent study (Gras *et al*., 2018), *SMZ* and its close homolog *SNZ* were positively regulated by nitrate and this was associated with delayed flowering in high N. However, their data indicated they are induced by an indirect route via the GA pathway (Gras *et al*., 2018), which did not change in our growth system. We believe that the nine NLPs encoded by the *A. thaliana* genome add great flexibility for tissue and affinity specificity for the individual NRE motifs. Low affinity for NRE motifs might become relevant, for example, when nitrate rises to concentrations that exceed those to which plants can easily adapt, leading to growth retardation, including a severe delay in flowering.

Interestingly, we found that the nitrate and T6P pathways converge at the same node within the flowering network. In addition to induction of *FT* in leaves, the T6P pathway activates *SPL3‐5* expression in the SAM, acting partly via a miR156‐dependent pathway and partly via a miR156‐independent pathway (Wahl *et al*., 2013). To date we cannot tell which of the *SPL*s is more sensitive to nitrate or T6P. It will also be interesting to learn whether the T6P and nitrate pathways converge at other important checkpoints as well, such as shown for yeast (Wilson *et al*., 2007) and plants (Figueroa *et al*., 2016) where post‐translational regulation of nitrate reductase strongly depends on the T6P signaling pathway. In this sense, residual TPS1 and T6P in the *35S::amiRTPS1* knockdown line might still be able to induce flowering in ON but no longer suffices to induce flowering in LN.

Our data reveal the crucial role of central players of the flowering time network at the SAM in the perception of soil N concentrations, which can only be overridden by an input of the T6P pathway in SD conditions or the photoperiod pathway in LD conditions. This highlights the importance of these pathways in the regulation of flowering time in response to the metabolic state of the plants. Taking this as a basis, new strategies for improving crop performance under nitrate‐limited conditions are possible.

## Author contributions

VW and AS conceived and designed the experiments. JJO, AS and VW performed all experiments, except for the main part of the plant work, generation of the NRE lines and the GUS stainings (CA), gathering of RNA *in situ* hybridization and flowering time data for NLPs (JvD), measurements of T6P concentrations (RF), analysis of the vegetative phase change and growth parameters (MAD), and generating and verifying *nlp* mutant lines (AK). JJO, AS and VW analyzed the data. VW wrote the paper with contributions from JJO and AS. All authors read and commented on the manuscript before submission.

## Supporting information

Please note: Wiley Blackwell are not responsible for the content or functionality of any Supporting Information supplied by the authors. Any queries (other than missing material) should be directed to the *New Phytologist* Central Office.

**Fig. S1** N‐limited *Arabidopsis thaliana* wild‐type (Col‐0) plants display developmental changes but no response to stress.
**Fig. S2** Leaf initiation rate *Arabidopsis thaliana* (Col‐0) plants.
**Fig. S3** The vegetative phase transition is not changed in nitrate‐limited *Arabidopsis thaliana* plants.
**Fig. S4** Expression analyses of relevant genes in gibberelic acid (GA) signaling in *Arabidopsis thaliana*.
**Fig. S5** Analyses of the components of the trehalose 6‐phosphate (T6P), age and photoperiod pathways in *Arabidopsis thaliana*.
**Fig. S6** Expression analyses of nitrate assimilation genes at the shoot apical meristem (SAM) in *Arabidopsis thaliana*.
**Table S1** Oligonucleotides used in this study.
**Table S2** Flowering time data of experiments described in this study.
**Table S3** Analyses of upstream intergenic regions of selected flowering time gene loci.
**Table S4** List of genes associated with the regulation of flowering time analyzed for Table S3.

## References

[nph15812-bib-0001] Abe M , Kobayashi Y , Yamamoto S , Daimon Y , Yamaguchi A , Ikeda Y , Ichinoki H , Notaguchi M , Goto K , Araki T . 2005 FD, a bZIP protein mediating signals from the floral pathway integrator FT at the shoot apex. Science 309: 1052–1056. 16099979 10.1126/science.1115983

[nph15812-bib-0002] Blumel M , Dally N , Jung C . 2015 Flowering time regulation in crops‐what did we learn from Arabidopsis? Current Opinion in Biotechnology 32: 121–129. 25553537 10.1016/j.copbio.2014.11.023

[nph15812-bib-0003] Castaings L , Camargo A , Pocholle D , Gaudon V , Texier Y , Boutet‐Mercey S , Taconnat L , Renou JP , Daniel‐Vedele F , Fernandez E *et al* 2009 The nodule inception‐like protein 7 modulates nitrate sensing and metabolism in *Arabidopsis* . The Plant Journal 57: 426–435. 18826430 10.1111/j.1365-313X.2008.03695.x

[nph15812-bib-0004] Castro Marin I , Loef I , Bartetzko L , Searle I , Coupland G , Stitt M , Osuna D . 2011 Nitrate regulates floral induction in *Arabidopsis*, acting independently of light, gibberellin and autonomous pathways. Planta 233: 539–552. 21113723 10.1007/s00425-010-1316-5 PMC3043248

[nph15812-bib-0005] Chardin C , Girin T , Roudier F , Meyer C , Krapp A . 2014 The plant RWP‐RK transcription factors: key regulators of nitrogen responses and of gametophyte development. Journal of Experimental Botany 65: 5577–5587. 24987011 10.1093/jxb/eru261

[nph15812-bib-0006] Corbesier L , Bernier G , Perilleux C . 2002 C : N ratio increases in the phloem sap during floral transition of the long‐day plants *Sinapis alba* and *Arabidopsis thaliana* . Plant and Cell Physiology 43: 684–688. 12091723 10.1093/pcp/pcf071

[nph15812-bib-0007] Cross JM , von Korff M , Altmann T , Bartzetko L , Sulpice R , Gibon Y , Palacios N , Stitt M . 2006 Variation of enzyme activities and metabolite levels in 24 *Arabidopsis* accessions growing in carbon‐limited conditions. Plant Physiology 142: 1574–1588. 17085515 10.1104/pp.106.086629 PMC1676042

[nph15812-bib-0008] D'Aloia M , Bonhomme D , Bouche F , Tamseddak K , Ormenese S , Torti S , Coupland G , Perilleux C . 2011 Cytokinin promotes flowering of *Arabidopsis* via transcriptional activation of the FT paralogue TSF. The Plant Journal 65: 972–979. 21205031 10.1111/j.1365-313X.2011.04482.x

[nph15812-bib-0009] Figueroa CM , Feil R , Ishihara H , Watanabe M , Kolling K , Krause U , Hohne M , Encke B , Plaxton WC , Zeeman SC *et al* 2016 Trehalose 6‐phosphate coordinates organic and amino acid metabolism with carbon availability. The Plant Journal 85: 410–423. 26714615 10.1111/tpj.13114

[nph15812-bib-0010] Gibon Y , Blaesing OE , Hannemann J , Carillo P , Hohne M , Hendriks JH , Palacios N , Cross J , Selbig J , Stitt M . 2004 A Robot‐based platform to measure multiple enzyme activities in *Arabidopsis* using a set of cycling assays: comparison of changes of enzyme activities and transcript levels during diurnal cycles and in prolonged darkness. Plant Cell 16: 3304–3325. 15548738 10.1105/tpc.104.025973 PMC535875

[nph15812-bib-0011] Good AG , Beatty PH . 2011 Fertilizing nature: a tragedy of excess in the commons. PLoS Biology 9: e1001124. 21857803 10.1371/journal.pbio.1001124 PMC3156687

[nph15812-bib-0012] Gowri G , Kenis JD , Ingemarsson B , Redinbaugh MG , Campbell WH . 1992 Nitrate reductase transcript is expressed in the primary response of maize to environmental nitrate. Plant Molecular Biology 18: 55–64. 1731978 10.1007/BF00018456

[nph15812-bib-0013] Gras DE , Vidal EA , Undurraga SF , Riveras E , Moreno S , Dominguez‐Figueroa J , Alabadi D , Blazquez MA , Medina J , Gutierrez RA . 2018 SMZ/SNZ and gibberellin signaling are required for nitrate‐elicited delay of flowering time in *Arabidopsis thaliana* . Journal of Experimental Botany 69: 619–631. 29309650 10.1093/jxb/erx423 PMC5853263

[nph15812-bib-0014] Guan P , Ripoll JJ , Wang R , Vuong L , Bailey‐Steinitz LJ , Ye D , Crawford NM . 2017 Interacting TCP and NLP transcription factors control plant responses to nitrate availability. Proceedings of the National Academy of Sciences, USA 114: 2419–2424. 10.1073/pnas.1615676114 PMC5338533 28202720

[nph15812-bib-0015] Hartmann U , Hohmann S , Nettesheim K , Wisman E , Saedler H , Huijser P . 2000 Molecular cloning of SVP: a negative regulator of the floral transition in *Arabidopsis* . The Plant Journal 21: 351–360. 10758486 10.1046/j.1365-313x.2000.00682.x

[nph15812-bib-0016] Hendriks JH , Kolbe A , Gibon Y , Stitt M , Geigenberger P . 2003 ADP‐glucose pyrophosphorylase is activated by posttranslational redox‐modification in response to light and to sugars in leaves of *Arabidopsis* and other plant species. Plant Physiology 133: 838–849. 12972664 10.1104/pp.103.024513 PMC219057

[nph15812-bib-0017] Hyun Y , Richter R , Vincent C , Martinez‐Gallegos R , Porri A , Coupland G . 2016 Multi‐layered regulation of SPL15 and cooperation with SOC1 integrate endogenous flowering pathways at the *Arabidopsis* shoot meristem. Developmental Cell 37: 254–266. 27134142 10.1016/j.devcel.2016.04.001

[nph15812-bib-0018] Jang S , Marchal V , Panigrahi KC , Wenkel S , Soppe W , Deng XW , Valverde F , Coupland G . 2008 *Arabidopsis* COP1 shapes the temporal pattern of CO accumulation conferring a photoperiodic flowering response. EMBO Journal 27: 1277–1288. 18388858 10.1038/emboj.2008.68 PMC2291449

[nph15812-bib-0019] Jang S , Torti S , Coupland G . 2009 Genetic and spatial interactions between *FT*,* TSF* and *SVP* during the early stages of floral induction in *Arabidopsis* . The Plant Journal 60: 614–625. 19656342 10.1111/j.1365-313X.2009.03986.x

[nph15812-bib-0020] Jung JH , Lee HJ , Ryu JY , Park CM . 2016 SPL3/4/5 integrate developmental aging and photoperiodic signals into the FT‐FD module in *Arabidopsis* flowering. Molecular Plant 9: 1647–1659. 27815142 10.1016/j.molp.2016.10.014

[nph15812-bib-0021] Kant S , Peng M , Rothstein SJ . 2011 Genetic regulation by NLA and microRNA827 for maintaining nitrate‐dependent phosphate homeostasis in *Arabidopsis* . PLoS Genetics 7: e1002021. 21455488 10.1371/journal.pgen.1002021 PMC3063762

[nph15812-bib-0022] Kazan K , Lyons R . 2016 The link between flowering time and stress tolerance. Journal of Experimental Botany 67: 47–60. 26428061 10.1093/jxb/erv441

[nph15812-bib-0023] Kim SG , Kim SY , Park CM . 2007 A membrane‐associated NAC transcription factor regulates salt‐responsive flowering via *FLOWERING LOCUS T* in *Arabidopsis* . Planta 226: 647–654. 17410378 10.1007/s00425-007-0513-3

[nph15812-bib-0024] Klein D , Morcuende R , Stitt M , Krapp A . 2000 Regulation of nitrate reductase expression in leaves by nitrate and nitrogen metabolism is completely overridden when sugars fall below a critical level. Plant, Cell & Environment 23: 863–871.

[nph15812-bib-0025] Konishi M , Yanagisawa S . 2010 Identification of a nitrate‐responsive *cis*‐element in the *Arabidopsis NIR1* promoter defines the presence of multiple *cis*‐regulatory elements for nitrogen response. The Plant Journal 63: 269–282. 20444232 10.1111/j.1365-313X.2010.04239.x

[nph15812-bib-0026] Konishi M , Yanagisawa S . 2011 Roles of the transcriptional regulation mediated by the nitrate‐responsive cis‐element in higher plants. Biochemical and Biophysical Research Communications 411: 708–713. 21777567 10.1016/j.bbrc.2011.07.008

[nph15812-bib-0027] Konishi M , Yanagisawa S . 2013 *Arabidopsis* NIN‐like transcription factors have a central role in nitrate signalling. Nature Communications 4: 1617. 10.1038/ncomms2621 23511481

[nph15812-bib-0028] Krapp A , Berthome R , Orsel M , Mercey‐Boutet S , Yu A , Castaings L , Elftieh S , Major H , Renou JP , Daniel‐Vedele F . 2011 *Arabidopsis* roots and shoots show distinct temporal adaptation patterns toward nitrogen starvation. Plant Physiology 157: 1255–1282. 21900481 10.1104/pp.111.179838 PMC3252138

[nph15812-bib-0029] Krapp A , David LC , Chardin C , Girin T , Marmagne A , Leprince AS , Chaillou S , Ferrario‐Mery S , Meyer C , Daniel‐Vedele F . 2014 Nitrate transport and signalling in *Arabidopsis* . Journal of Experimental Botany 65: 789–798. 24532451 10.1093/jxb/eru001

[nph15812-bib-0030] Krouk G , Ruffel S , Gutierrez RA , Gojon A , Crawford NM , Coruzzi GM , Lacombe B . 2011 A framework integrating plant growth with hormones and nutrients. Trends in Plant Science 16: 178–182. 21393048 10.1016/j.tplants.2011.02.004

[nph15812-bib-0031] Landrein B , Formosa‐Jordan P , Malivert A , Schuster C , Melnyk CW , Yang W , Turnbull C , Meyerowitz EM , Locke JCW , Jonsson H . 2018 Nitrate modulates stem cell dynamics in *Arabidopsis* shoot meristems through cytokinins. Proceedings of the National Academy of Sciences, USA 115: 1382–1387. 10.1073/pnas.1718670115 PMC5819446 29363596

[nph15812-bib-0032] Laubinger S , Marchal V , Le Gourrierec J , Wenkel S , Adrian J , Jang S , Kulajta C , Braun H , Coupland G , Hoecker U . 2006 *Arabidopsis* SPA proteins regulate photoperiodic flowering and interact with the floral inducer CONSTANS to regulate its stability. Development 133: 3213–3222. 16854975 10.1242/dev.02481

[nph15812-bib-0033] Lee JH , Yoo SJ , Park SH , Hwang I , Lee JS , Ahn JH . 2007 Role of *SVP* in the control of flowering time by ambient temperature in *Arabidopsis* . Genes & Development 21: 397–402. 17322399 10.1101/gad.1518407 PMC1804328

[nph15812-bib-0034] Liang G , He H , Yu D . 2012 Identification of nitrogen starvation‐responsive microRNAs in Arabidopsis thaliana. PLoS ONE 7: e48951. 23155433 10.1371/journal.pone.0048951 PMC3498362

[nph15812-bib-0035] Lim MH , Kim J , Kim YS , Chung KS , Seo YH , Lee I , Kim J , Hong CB , Kim HJ , Park CM . 2004 A new *Arabidopsis* gene, *FLK*, encodes an RNA binding protein with K homology motifs and regulates flowering time via *FLOWERING LOCUS C* . Plant Cell 16: 731–740. 14973162 10.1105/tpc.019331 PMC385284

[nph15812-bib-0036] Lin YL , Tsay YF . 2017 Influence of differing nitrate and nitrogen availability on flowering control in *Arabidopsis* . Journal of Experimental Botany 68: 2603–2609. 28369493 10.1093/jxb/erx053

[nph15812-bib-0037] Liu T , Li Y , Ren J , Qian Y , Yang X , Duan W , Hou X . 2013 Nitrate or NaCl regulates floral induction in *Arabidopsis thaliana* . Biologia 68: 215–222.

[nph15812-bib-0038] Livak KJ , Schmittgen TD . 2001 Analysis of relative gene expression data using real‐time quantitative PCR and the 2(‐Delta Delta C(T)) Method. Methods 25: 402–408. 11846609 10.1006/meth.2001.1262

[nph15812-bib-0039] Lunn JE , Feil R , Hendriks JH , Gibon Y , Morcuende R , Osuna D , Scheible WR , Carillo P , Hajirezaei MR , Stitt M . 2006 Sugar‐induced increases in trehalose 6‐phosphate are correlated with redox activation of ADPglucose pyrophosphorylase and higher rates of starch synthesis in *Arabidopsis thaliana* . Biochemical Journal 397: 139–148. 16551270 10.1042/BJ20060083 PMC1479759

[nph15812-bib-0040] Marchive C , Roudier F , Castaings L , Brehaut V , Blondet E , Colot V , Meyer C , Krapp A . 2013 Nuclear retention of the transcription factor NLP7 orchestrates the early response to nitrate in plants. Nature Communications 4: 1713. 10.1038/ncomms2650 23591880

[nph15812-bib-0041] Michaels SD , Amasino RM . 2001 Loss of *FLOWERING LOCUS C* activity eliminates the late‐flowering phenotype of *FRIGIDA* and autonomous pathway mutations but not responsiveness to vernalization. Plant Cell 13: 935–941. 11283346 10.1105/tpc.13.4.935 PMC135534

[nph15812-bib-0042] Michaels SD , Himelblau E , Kim SY , Schomburg FM , Amasino RM . 2005 Integration of flowering signals in winter‐annual *Arabidopsis* . Plant Physiology 137: 149–156. 15618421 10.1104/pp.104.052811 PMC548846

[nph15812-bib-0043] Noguero M , Lacombe B . 2016 Transporters involved in root nitrate uptake and sensing by *Arabidopsis* . Frontiers in Plant Science 7: 1391. 27708653 10.3389/fpls.2016.01391 PMC5030233

[nph15812-bib-0044] Nunes‐Nesi A , Carrari F , Gibon Y , Sulpice R , Lytovchenko A , Fisahn J , Graham J , Ratcliffe RG , Sweetlove LJ , Fernie AR . 2007 Deficiency of mitochondrial fumarase activity in tomato plants impairs photosynthesis via an effect on stomatal function. The Plant Journal 50: 1093–1106. 17461782 10.1111/j.1365-313X.2007.03115.x

[nph15812-bib-0045] Nunes‐Nesi A , Fernie AR , Stitt M . 2010 Metabolic and signaling aspects underpinning the regulation of plant carbon nitrogen interactions. Molecular Plant 3: 973–996. 20926550 10.1093/mp/ssq049

[nph15812-bib-0046] Pant BD , Musialak‐Lange M , Nuc P , May P , Buhtz A , Kehr J , Walther D , Scheible WR . 2009 Identification of nutrient‐responsive *Arabidopsis* and rapeseed microRNAs by comprehensive real‐time polymerase chain reaction profiling and small RNA sequencing. Plant Physiology 150: 1541–1555. 19465578 10.1104/pp.109.139139 PMC2705054

[nph15812-bib-0047] Rhoades MW , Reinhart BJ , Lim LP , Burge CB , Bartel B , Bartel DP . 2002 Prediction of plant microRNA targets. Cell 110: 513–520. 12202040 10.1016/s0092-8674(02)00863-2

[nph15812-bib-0048] Rideout JW , Raper CD Jr , Miner GS . 1992 Changes in ratio of soluble sugars and free amino nitrogen in the apical meristem during floral transition of tobacco. International Journal of Plant Sciences 153: 78–88. 11537504 10.1086/297008

[nph15812-bib-0049] Rosso MG , Li Y , Strizhov N , Reiss B , Dekker K , Weisshaar B . 2003 An *Arabidopsis thaliana* T‐DNA mutagenized population (GABI‐Kat) for flanking sequence tag‐based reverse genetics. Plant Molecular Biology 53: 247–259. 14756321 10.1023/B:PLAN.0000009297.37235.4a

[nph15812-bib-0050] Rowan DD , Cao M , Lin‐Wang K , Cooney JM , Jensen DJ , Austin PT , Hunt MB , Norling C , Hellens RP , Schaffer RJ *et al* 2009 Environmental regulation of leaf colour in red *35S:PAP1 Arabidopsis thaliana* . New Phytologist 182: 102–115. 19192188 10.1111/j.1469-8137.2008.02737.x

[nph15812-bib-0051] Scheible WR , Gonzalez‐Fontes A , Lauerer M , Muller‐Rober B , Caboche M , Stitt M . 1997 Nitrate acts as a signal to induce organic acid metabolism and repress starch metabolism in tobacco. Plant Cell 9: 783–798. 12237366 10.1105/tpc.9.5.783 PMC156956

[nph15812-bib-0052] Schmid M , Uhlenhaut NH , Godard F , Demar M , Bressan R , Weigel D , Lohmann JU . 2003 Dissection of floral induction pathways using global expression analysis. Development 130: 6001–6012. 14573523 10.1242/dev.00842

[nph15812-bib-0053] Schwab R , Palatnik JF , Riester M , Schommer C , Schmid M , Weigel D . 2005 Specific effects of microRNAs on the plant transcriptome. Developmental Cell 8: 517–527. 15809034 10.1016/j.devcel.2005.01.018

[nph15812-bib-0054] Seligman K , Saviani EE , Oliveira HC , Pinto‐Maglio CA , Salgado I . 2008 Floral transition and nitric oxide emission during flower development in *Arabidopsis thaliana* is affected in nitrate reductase‐deficient plants. Plant and Cell Physiology 49: 1112–1121. 18540030 10.1093/pcp/pcn089

[nph15812-bib-0055] Shibata H , Branquinho C , McDowell WH , Mitchell MJ , Monteith DT , Tang J , Arvola L , Cruz C , Cusack DF , Halada L *et al* 2015 Consequence of altered nitrogen cycles in the coupled human and ecological system under changing climate: the need for long‐term and site‐based research. Ambio 44: 178–193. 25037589 10.1007/s13280-014-0545-4 PMC4357624

[nph15812-bib-0056] Smil V . 1999 Nitrogen in crop production: an account of global flows. Global Biogeochemical Cycles 13: 647–662.

[nph15812-bib-0057] Srikanth A , Schmid M . 2011 Regulation of flowering time: all roads lead to Rome. Cellular and Molecular Life Sciences 68: 2013–2037. 21611891 10.1007/s00018-011-0673-y PMC11115107

[nph15812-bib-0058] Stitt M , Lilley RM , Gerhardt R , Heldt HW . 1989 Metabolite levels in specific cells and subcellular compartments of plant leaves. Methods in Enzymology 174: 518–552.

[nph15812-bib-0059] Takeno K . 2016 Stress‐induced flowering: the third category of flowering response. Journal of Experimental Botany 67: 4925–4934. 27382113 10.1093/jxb/erw272

[nph15812-bib-0060] Telfer A , Bollman KM , Poethig RS . 1997 Phase change and the regulation of trichome distribution in *Arabidopsis thaliana* . Development 124: 645–654. 9043079 10.1242/dev.124.3.645

[nph15812-bib-0061] Tischner R . 2001 Nitrate uptake and reduction in higher and lower plants. Plant, Cell & Environment 23: 1005–1024.

[nph15812-bib-0062] Tocquin P , Corbesier L , Havelange A , Pieltain A , Kurtem E , Bernier G , Perilleux C . 2003 A novel high efficiency, low maintenance, hydroponic system for synchronous growth and flowering of *Arabidopsis thaliana* . BMC Plant Biology 3: 2. 12556248 10.1186/1471-2229-3-2 PMC150571

[nph15812-bib-0063] Torti S , Fornara F , Vincent C , Andres F , Nordstrom K , Gobel U , Knoll D , Schoof H , Coupland G . 2012 Analysis of the *Arabidopsis* shoot meristem transcriptome during floral transition identifies distinct regulatory patterns and a leucine‐rich repeat protein that promotes flowering. Plant Cell 24: 444–462. 22319055 10.1105/tpc.111.092791 PMC3315226

[nph15812-bib-0064] Tschoep H , Gibon Y , Carillo P , Armengaud P , Szecowka M , Nunes‐Nesi A , Fernie AR , Koehl K , Stitt M . 2009 Adjustment of growth and central metabolism to a mild but sustained nitrogen‐limitation in *Arabidopsis* . Plant, Cell & Environment 32: 300–318. 10.1111/j.1365-3040.2008.01921.x 19054347

[nph15812-bib-0065] Vidal EA , Moyano TC , Canales J , Gutierrez RA . 2014 Nitrogen control of developmental phase transitions in *Arabidopsis thaliana* . Journal of Experimental Botany 65: 5611–5618. 25129132 10.1093/jxb/eru326

[nph15812-bib-0066] Wahl V , Ponnu J , Schlereth A , Arrivault S , Langenecker T , Franke A , Feil R , Lunn JE , Stitt M , Schmid M . 2013 Regulation of flowering by trehalose‐6‐phosphate signaling in *Arabidopsis thaliana* . Science 339: 704–707. 23393265 10.1126/science.1230406

[nph15812-bib-0067] Wan CY , Wilkins TA . 1994 A modified hot borate method significantly enhances the yield of high‐quality RNA from cotton (*Gossypium hirsutum* L.). Analytical Biochemistry 223: 7–12. 7535022 10.1006/abio.1994.1538

[nph15812-bib-0068] Wang JW . 2014 Regulation of flowering time by the miR156‐mediated age pathway. Journal of Experimental Botany 65: 4723–4730. 24958896 10.1093/jxb/eru246

[nph15812-bib-0069] Wang JW , Czech B , Weigel D . 2009 miR156‐regulated *SPL* transcription factors define an endogenous flowering pathway in *Arabidopsis thaliana* . Cell 138: 738–749. 19703399 10.1016/j.cell.2009.06.014

[nph15812-bib-0070] Wang JW , Schwab R , Czech B , Mica E , Weigel D . 2008 Dual effects of miR156‐targeted *SPL* genes and *CYP78A5/KLUH* on plastochron length and organ size in *Arabidopsis thaliana* . Plant Cell 20: 1231–1243. 18492871 10.1105/tpc.108.058180 PMC2438454

[nph15812-bib-0071] Wang R , Farrona S , Vincent C , Joecker A , Schoof H , Turck F , Alonso‐Blanco C , Coupland G , Albani MC . 2009 *PEP1* regulates perennial flowering in *Arabis alpina* . Nature 459: 423–427. 19369938 10.1038/nature07988

[nph15812-bib-0072] Ward MH , Jones RR , Brender JD , de Kok TM , Weyer PJ , Nolan BT , Villanueva CM , van Breda SG . 2018 Drinking water nitrate and human health: an updated review. International Journal of Environmental Research and Public Health 15: 1557–1588. 10.3390/ijerph15071557 PMC6068531 30041450

[nph15812-bib-0073] Weber K , Burow M . 2018 Nitrogen – essential macronutrient and signal controlling flowering time. Physiologia Plantarum 162: 251–260. 29095491 10.1111/ppl.12664

[nph15812-bib-0074] Weigel D , Alvarez J , Smyth DR , Yanofsky MF , Meyerowitz EM . 1992 *LEAFY* controls floral meristem identity in *Arabidopsis* . Cell 69: 843–859. 1350515 10.1016/0092-8674(92)90295-n

[nph15812-bib-0075] Wilson RA , Jenkinson JM , Gibson RP , Littlechild JA , Wang ZY , Talbot NJ . 2007 Tps1 regulates the pentose phosphate pathway, nitrogen metabolism and fungal virulence. EMBO Journal 26: 3673–3685. 17641690 10.1038/sj.emboj.7601795 PMC1949003

[nph15812-bib-0076] Winter D , Vinegar B , Nahal H , Ammar R , Wilson GV , Provart NJ . 2007 An “Electronic Fluorescent Pictograph” browser for exploring and analyzing large‐scale biological data sets. PLoS ONE 2: e718. 17684564 10.1371/journal.pone.0000718 PMC1934936

[nph15812-bib-0077] Xu M , Hu T , Zhao J , Park MY , Earley KW , Wu G , Yang L , Poethig RS . 2016 Developmental functions of miR156‐regulated *SQUAMOSA PROMOTER BINDING PROTEIN‐LIKE* (*SPL*) genes in *Arabidopsis thaliana* . PLoS Genetics 12: e1006263. 27541584 10.1371/journal.pgen.1006263 PMC4991793

[nph15812-bib-0078] Yadav UP , Ivakov A , Feil R , Duan GY , Walther D , Giavalisco P , Piques M , Carillo P , Hubberten HM , Stitt M *et al* 2014 The sucrose‐trehalose 6‐phosphate (Tre6P) nexus: specificity and mechanisms of sucrose signalling by Tre6P. Journal of Experimental Botany 65: 1051–1068. 24420566 10.1093/jxb/ert457 PMC3935566

[nph15812-bib-0079] Yoo SK , Chung KS , Kim J , Lee JH , Hong SM , Yoo SJ , Yoo SY , Lee JS , Ahn JH . 2005 *CONSTANS* activates *SUPPRESSOR OF OVEREXPRESSION OF CONSTANS 1* through *FLOWERING LOCUS T* to promote flowering in *Arabidopsis* . Plant Physiology 139: 770–778. 16183837 10.1104/pp.105.066928 PMC1255994

[nph15812-bib-0080] Yu LH , Wu J , Tang H , Yuan Y , Wang SM , Wang YP , Zhu QS , Li SG , Xiang CB . 2016 Overexpression of *Arabidopsis NLP7* improves plant growth under both nitrogen‐limiting and ‐sufficient conditions by enhancing nitrogen and carbon assimilation. Scientific Reports 6: 27795. 27293103 10.1038/srep27795 PMC4904239

[nph15812-bib-0081] Yuan S , Zhang ZW , Zheng C , Zhao ZY , Wang Y , Feng LY , Niu G , Wang CQ , Wang JH , Feng H *et al* 2016 *Arabidopsis* cryptochrome 1 functions in nitrogen regulation of flowering. Proceedings of the National Academy of Sciences, USA 113: 7661–7666. 10.1073/pnas.1602004113 PMC4941442 27325772

