# Supplementary material for: Nitrate acts at the Arabidopsis thaliana shoot apical meristem to regulate flowering time
Source: New Phytol. 2019 Apr 17;223(2):814–27. doi: 10.1111/nph.15812 (PMC6618062; doi:10.1111/nph.15812)
Supplement: Supplementary file 1 — Fig. S1 N‐limited Arabidopsis thaliana wild‐type (Col‐0) plants display developmental changes but no response to stress. Fig. S2 Leaf initiation rate Arabidopsis thaliana (Col‐0) plants. Fig. S3 The vegetative phase transition is not changed in nitrate‐limited Arabidopsis thaliana plants. Fig. S4 Expression analyses of relevant genes in gibberelic acid (GA) signaling in Arabidopsis thaliana. Fig. S5 Analyses of the components of the trehalose 6‐phosphate (T6P), age and photoperiod pathways in Arabidopsis thaliana. Fig. S6 Expression analyses of nitrate assimilation genes at the shoot apical meristem (SAM) in Arabidopsis thaliana. Table S1 Oligonucleotides used in this study. Table S2 Flowering time data of experiments described in this study. Table S3 Analyses of upstream intergenic regions of selected flowering time gene loci. Table S4 List of genes associated with the regulation of flowering time analyzed for Table S3. [file NPH-223-814-s001.pdf]

New Phytologist **Supporting Information**

Nitrate acts at the *Arabidopsis* shoot apical meristem to regulate flowering time

Authors Justyna Jadwiga Olas, Judith Van Dingenen, Christin Abel, Magdalena Anna Działo, Regina Feil, Anne Krapp, Armin Schlereth, Vanessa Wahl

Article acceptance date: 15 March 2019.

The following supporting information is available for this article:

Fig. S1 N-limited *Arabidopsis thaliana* wild-type (Col-0) plants display developmental changes but no response to stress.

Fig. S2 Leaf initiation rate of *Arabidopsis thaliana* (Col-0) plants.

Fig. S3 The vegetative phase transition is not changed in nitrate-limited *Arabidopsis thaliana* plants.

Fig. S4 Expression analyses of relevant genes in gibberelic acid (GA) signaling in *Arabidopsis thaliana*.

Fig. S5 Analyses of components of the trehalose 6-phosphate (T6P), age and photoperiod pathways in *Arabidopsis thaliana*.

Fig. S6 Expression analyses of nitrate assimilation genes at the shoot apical meristem (SAM) in *Arabidopsis thaliana*.

Table S1 Oligonucleotides used in this study.

Table S2 Flowering time data of experiments described in this study.

Table S3 Analyses of upstream intergenic regions of selected flowering time gene loci.

Table S4 List of genes associated with the regulation of flowering time analyzed for Table S3.

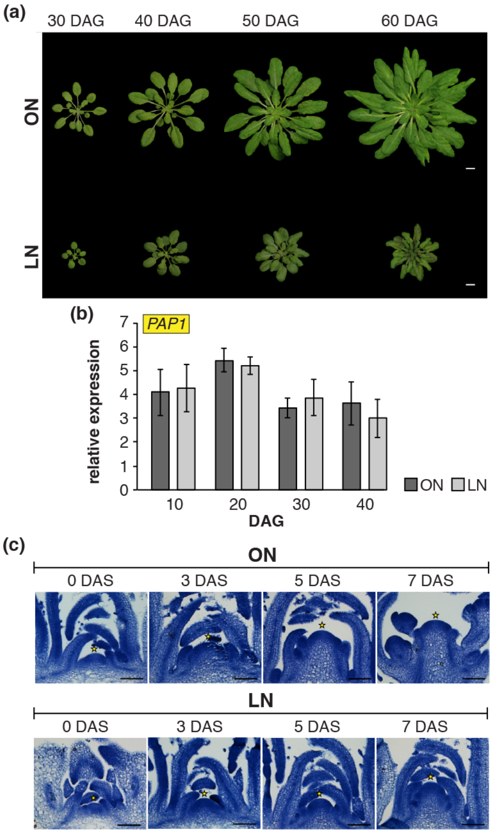

Fig. S1 N-limited *Arabidopsis thaliana* wild-type (Col-0) plants display developmental changes but no response to stress. (**a**) Rosette phenotype of wild-type plants grown in both nitrogen (N) regimes in short days (SD, 8h light/16h dark) at 30, 40, 50 and 60 days after germination (DAG). Plants grown in the limited N (LN) soil did not display visual symptoms of stress such as accelerated senescence and adapted to the LN treatment. (**b**) Expression analysis of the stress marker gene *ARABIDOPSIS THALIANA PRODUCTION OF ANTHOCYANIN PIGMENT 1* (*PAP1*, At1g56650, Rowan *et al.*, 2009) measured by qRT-PCR in whole rosettes of plants grown in SD at 10, 20, 30 and 40 DAG and harvested at the end of the day. (**c**) Toluidine blue stained longitudinal sections through apices of 30-day-old SD-grown plants transferred to long days (LD) for 3, 5 and 7 days, in order to synchronously induce photoperiod-dependent flowering. DAS, days after the shift. Asterisks (c) indicate meristem summit. Error bars denote s.d. No statistically significant difference (Student's t-test) was found between ON and LN grown plants. Scale bars: 1cm (a) and 100µm (c).

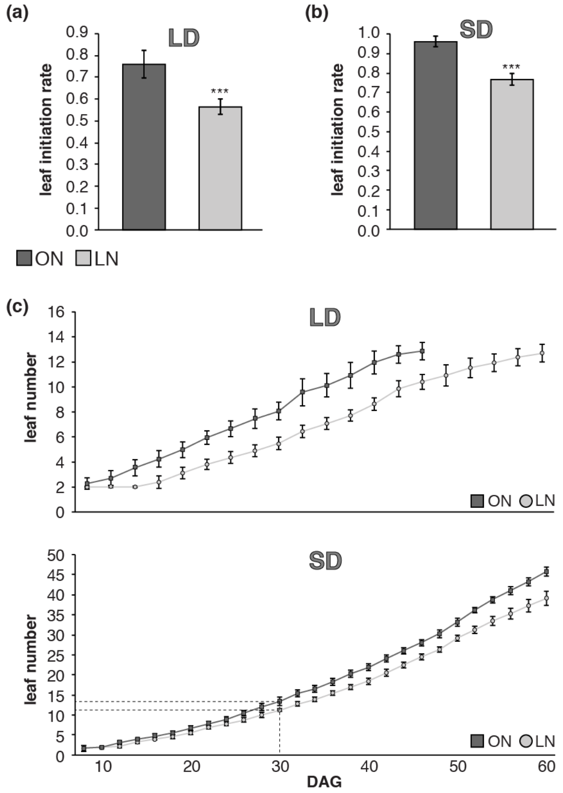

Fig. S2 Leaf initiation rate *Arabidopsis thaliana* (Col-0) plants. (**a-c**) When analyzing plants grown in the two nitrogen (N) regimes, a reduced leaf initiation rate was found in plants grown on LN in long days (LD) (a, c) and short days (SD) (b, c), both when dividing the total number of leaves by the days to bolting (DTB; a, b, Supporting Information Table S2 for corresponding data) and by counting the appearance of 2mm-sized leaves throughout vegetative development (c). Dashed line in (c) indicates the number of juvenile leaves at the time plants were transferred into LD in a SD to LD shift experiment. This demonstrates that plants grown in both regimes were equally competent to induce flowering at the time of the shift as the vegetative phase change occurs before 30 DAG (for details on juvenile leaf numbers see Fig. S3). ON, optimal N soil; LN, limited N soil; DAG, days after the germination. Error bars denote s.d., statistical significance between ON and LN was calculated using the Student's t-test: *** $p \leq 0.001$.

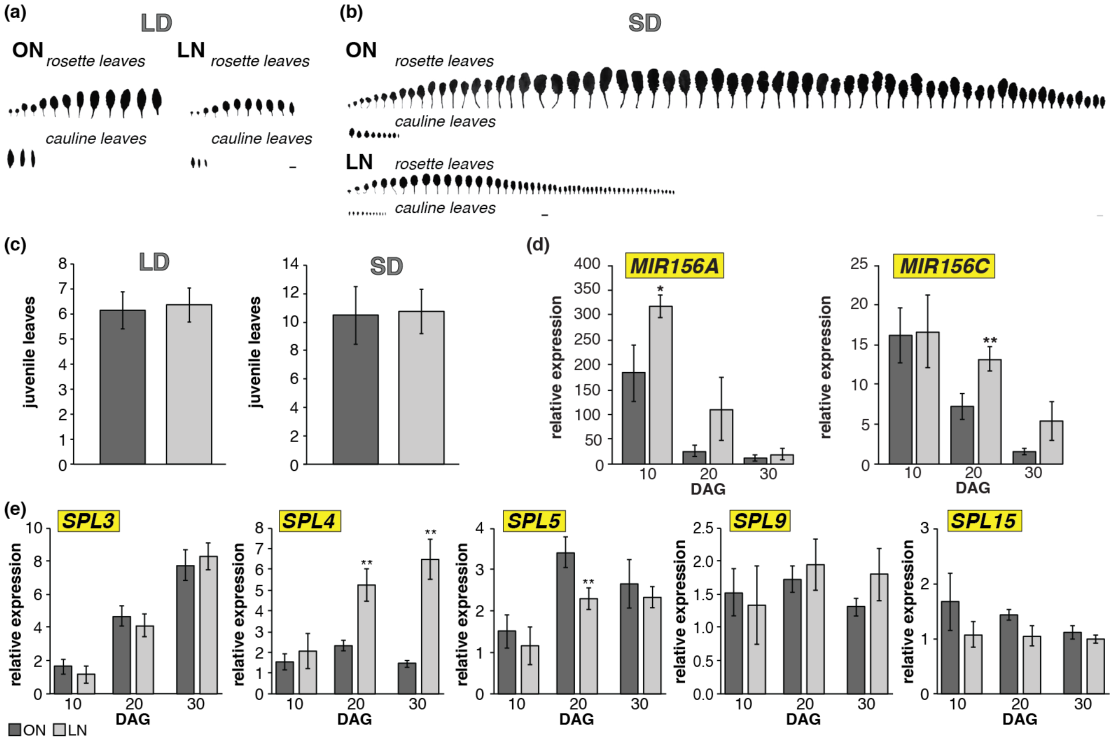

Fig. S3 The vegetative phase transition is not changed in nitrate-limited *Arabidopsis thaliana* plants. (**a, b**) Leaf imprints from plants in long days (LD) (a) and in short days (SD) (b). (**c**) Similar numbers of juvenile leaves (without abaxial trichomes) also indicated no effect of the nitrate treatment on the transition from the juvenile to the adult phase in LD (16h light/8h dark; ON: 6.15 ± 0.75, n=20, LN: 6.35 ± 0.67, n=20) and in SD (8h light/16h dark; ON: 10.47 ± 2.03, n=17, LN: 10.75 ± 1.59, n=20). (**d, e**) Transcript levels of *MIR156A* and *MIR156C* (d) and *SQUAMOSA PROMOTER BINDING LIKE PROTEINs* (*SPL*s) (e) determined in rosettes of ON and LN plants grown in SD (8h light/16h dark) and harvested at the end of the day by qRT-PCR. ON, optimal N soil; LN, limited N soil; DAG, days after germination. Error bars denote s.d., statistical significance between ON and LN was calculated using the Student's t-test: * $p < 0.05$; ** $p < 0.01$. Scale bars: 1cm (a, b).

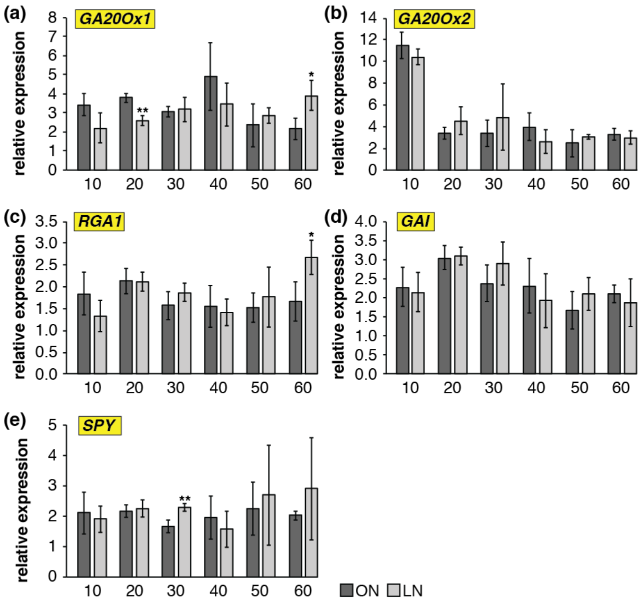

Fig. S4 Expression analyses of relevant genes in gibberelic acid (GA) signaling in *Arabidopsis thaliana*. (**a-e**) Transcript levels were obtained by qRT-PCR of (a) *GIBBERELLIN 20-OXIDASE 1* (*GA20Ox1*, At4g25420), (b) *GIBBERELLIN 20-OXIDASE 2* (*GA20Ox2*, At5g51810), (c) *REPRESSOR OF GA 1* (*RGA1*, At2g01570), (d) *GIBBERELLIC ACID INSENSITIVE* (*GAI*, At1g14920) and (e) *SPINDLY* (*SPY*, At3g11540) in rosettes of short days (SD) grown plants harvested at the end of the day as a readout for signaling via the GA pathway (Mutasa-Gottgens & Hedden, 2009). This data does not support an interaction between N and GA signaling in the regulation of flowering time. DAG, days after germination. Error bars denote s.d., the statistical significance between ON and LN was calculated using the Student's t-test: * $p < 0.05$, ** $p < 0.01$.

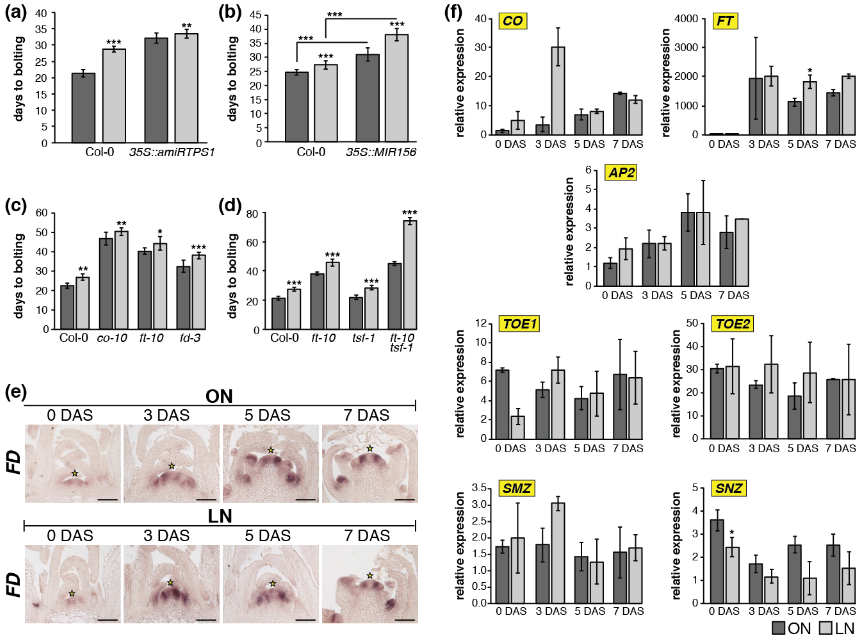

Fig. S5 Analyses of the components of the trehalose 6-phosphate (T6P), age and photoperiod pathways in *Arabidopsis thaliana*. (**a-d**) Determining flowering time of the T6P (a), age (b) and photoperiod pathways (c-d). The flowering phenotype of the mutants demonstrates that the T6P, age and photoperiod pathways act at least partially independent of nitrate-dependent flowering. (**e-f**) Transcript analyses of components of the photoperiod pathway at the shoot apical meristem (SAM) of wild-type plants by RNA *in situ* hybridization using *FLOWERING LOCUS D* (*FD*, At4g35900) as a probe (e) and in the leaves by qRT-PCR (f) of *CONSTANS* (*CO*, At5g15840), *FLOWERING LOCUS T* (*FT*, AT1g65480) and repressors of *FT* (*APETALA 2*, *AP2*, At4g36920; *TARGET OF EAT 1*, *TOE1*, At2g28550; *TOE2*, At5g60120; *SCHLAFMÜTZE*, *SMZ*, At3g54990; *SCHNARCHZAPFEN*, *SNZ*, At2g39250) demonstrated that changes in components of the photoperiod pathway do not explain the delayed flowering phenotype observed when plants are grown on LN soil. Samples were harvested at the end of the day. LN, limited N soil; DAS, days after shift to long days. Asterisks (e) indicate meristem summit. Scale bars: 100µm (e). Error bars denote s.d., the statistical significance was calculated using the Student's t-test: * $p < 0.05$, ** $p < 0.01$, *** $p < 0.001$.

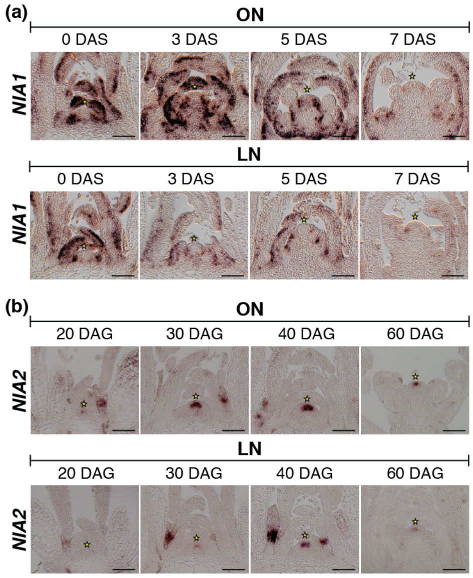

Fig. S6 Expression analyses of nitrate assimilation genes at the shoot apical meristem (SAM) in *Arabidopsis thaliana*. (**a-b**) RNA *in situ* hybridization on longitudinal sections through apices of plants grown in the two nitrogen (N) regimes using specific probes against *NITRATE REDUCTASE 1* (*NIA1,* At1g77760, a) and *NITRATE REDUCTASE 2* (*NIA2,* At1g37130, b). Much weaker signal of *NIA1* and *NIA2* was detected at the SAM of LN grown plants compared to ON plants. Plants were grown in a short day (SD)-shift experiment (a) or continuously in SD (b). ON, optimal N soil; LN, limited N soil; DAS, days after the shift to long days; DAG, days after germination. Asterisks (a, b) indicate meristem summit. Scale bars: 100µm (a, b).

Table S1 Oligonucleotides used in this study.

Gene (AGI)	Oligo	Sequence (5'→3')	Product lengths (bp)
**Oligonucleotides used for qRT-PCR**			
TUB2	P-344	GAGCCTTACAACGCTACTCTGTCTGTC	167
At5g62690	P-345	ACACCAGACATAGTAGCAGAAATCAAG	
SAND	P-346	AACTCTATGCAGCATTTGATCCACT	61
At2g28390	P-347	TGATTGCATATCTTTATCGCCATC	
UBI10	P-348	CACACTCCACTTGGTCTTGCGT	71
At4g05320	P-349	TGGTCTTTCCGGTGAGAGTCTTCA	
PDF2	P-350	TAACGTGGCCAAAATGATG	61
At1g13320	P-351	GTTCTCCACAACCGCTTGGT	
CO	P-370	AACAGCTTCACACCCAAGAACG	53
At5g15840	P-371	GGTCAGGTTGTTGCTCTACTGTCC	
FT	P-414	TGGAACAACCTTTGGCAATGAG	71
At1g65480	P-415	CGACACGATGAATTCCTGCAG	
LFY	P-434	AGTTCCTTCTTCAGGTCCAGACAATTG	171
At5g61850	P-435	CTTCTTCGTCTAGGCAGTGGAGAGCGT	
SOC1	P-532	TTGAGCAGCTCAAGCAAAAGGA	68
At2g45660	P-533	TCCCCACTTTTCAGAGAGCTTCTC	
SPL3	P-544	GAGTTTGTCAGGTCGAGAGTTGTACC	74
At2g33810	P-545	GCAGACTTTGTGTCGTTTGTGGT	
SPL4	P-546	AATGGTCAGGTGGTGATGCAG	61
At1g53160	P-547	GCATAGGAAGTGTCATCTCTACCCTT	
SPL5	P-548	CAGCAGGTTTCATGAGCTACCAG	107
At3g15270	P-549	CAAAACTGTCACCAGAGATCTTCCTC	
NIA1	NIA1-2708	CTGAAGGTTTGGAAGGCCAATC	60
AT1G77760	NIA1-2767	ACTGAATCATAGGCGGTGGTCC	
NIA2	NIA2-2637	TTGGTACGTAGTGGAATCAGCTAAGG	51
AT1G37130	NIA2-2687	AACCCGGTACTGTATGCCCAAC	
GAI	P-430	GGTGAGGGTTATCGGGTGGA	71
At1g14920	P-431	GCTATGAGCGGTCGTGTGTG	
SPY	P-552	GACCATCAAGAGTCAGCGTTACTG	71
At3g11540	P-553	AGGTACAGGAGCAGAACCATTGG	
GA20Ox1	P-426	CGGCGATACTTTCATGGCTCTAT	95
At4g25420	P-427	GCAAGTGATTTCCTCTCGCTCTC	
GA20Ox2	P-428	AAGAGCTGTTTGCATAGAGCGGT	68
At5g51810	P-429	TCACCACTTTGTCTTTCTTCGGAC	
**Oligonucleotides used for cloning**			
FD	P-0317	ATGTTGTCATCAGCTAAGCATC	
At4g35900	P-0318	TCAAAATGGAGCTGTGGAAGAC	
NIA1	P-0313	ATGGCGACCTCCGTCGATAAC	
At1g77760	P-0314	CTAGAAGATTAAGAGATCCTCC	
NIA2	P-0315	ATGGCGGCCTCTGTAGATAATCG	
At1g37130	P-0316	CTAGAATATCAAGAAATCCTCC	
NLP6	P-0628	ATGGAACTTGACGACTTGGATC	
At1g64530	P-0629	TCACAAGCACATCATAGTTTCCTC	
NLP7	P-0630	ATGTGCGAGCCCGATGATAATTC	
At4g24020	P-0631	TCACAATTCTCCAGTGCTCTCG	

Gene (AGI)	Oligo	Sequence (5'→3')	Product lengths (bp)
		**Oligonucleotides used for genotyping**	
TSF	G-6735	ATGACTTTGTGGTCAGAGTTAG	
At4g20370	G-6736	AAGTTAAAGATATGGTGCAGTG	
tsf-1	P-207	ATTTTGCCGATTTCGGAAC	
At4g20370	G-6736	AAGTTAAAGATATGGTGCAGTG	
FT	P-0231	ATATTGATGAATCTCTGTTGTGG	
At1g65480	P-0266	TTGATGCATCGCACTCTCGAAG	
ft-10	P-0266	TTGATGCATCGCACTCTCGAAG	
At1g65480	G-2081	CCCATTTGGACGTGAATGTAGACAC	
SOC1	P-0302	AGTTTCCATTACAGACTTATAG	
At2g45660	P-0299	AACTCATTTAAACACACCTCTC	
soc1-6	P-0207	ATTTTGCCGATTTCGGAAC	
At2g45660	P-0299	AACTCATTTAAACACACCTCTC	
NLP6	P-1056	TTTGCCTGTTTTTGAACCATC	
At1g64530	P-1057	TCCTGATTGGTTCAAGTTTCG	
nlp6-2	P-1057	TCCTGATTGGTTCAAGTTTCG	
At1g64530	P-0207	ATTTTGCCGATTTCGGAAC	
NLP7	P-0661	ACTCTTCTCCATCAGCAGCTGAG	
At4g24020	P-0662	CCTCAATATAACCGGCAAGGCA	
nlp7-1	P-0661	ACTCTTCTCCATCAGCAGCTGAG	
At4g24020	P-0207	ATTTTGCCGATTTCGGAAC	

Table S2 Flowering time data of experiments described in this study.

Plant line/Experiment	ON			LN		
	**DTB**	TLN	n	**DTB**	TLN	n
**Experiment 1 (long days)**						
Col-0 (wild type)	**21.3 ± 1.1**	16.2 ± 1.1	20	**28.7 ± 0.9**$^{(+)}$	16.2 ± 1.2	20
soc1-6	**29.6 ± 1.2**	29.8 ± 1.7	18	**30.5 ± 0.6**$^{(-)}$	27.6 ± 2.0	18
35S::amiRTPS1	**32.1 ± 1.5**	27.5 ± 2.3	19	**33.4 ± 1.3**$^{(+)}$	21.8 ± 1.8	17
**Experiment 2 (short days)**						
Col-0 (wild type)	**66.2 ± 1.6**	63.6 ± 2.3	16	**81.1 ± 4.3**$^{(+)}$	62.1 ± 2.6	18
soc1-6	**87.6 ± 5.0**	88.6 ± 2.1	16	**88.5 ± 4.7**$^{(-)}$	67.9 ± 3.1	15
35S::amiRTPS1	**76.5 ± 3.7**	59.3 ± 2.2	15	**n.f.**	n.f.	20
**Experiment 3 (long days)**						
Col-0 (wild type)	**21.3 ± 1.5**	16.1 ± 1.0	15	**27.3 ± 1.3**$^{(+)}$	17.4 ± 1.3	15
tsf-1	**22.0 ± 1.6**	16.3 ± 1.2	20	**28.5 ± 1.4**$^{(+)}$	17.8 ± 1.2	20
ft-10	**38.1 ± 1.1**	42.4 ± 1.4	15	**45.7 ± 2.5**$^{(+)}$	38.9 ± 2.9	13
ft-10;tsf-1	**44.9 ± 1.4**	59.7 ± 1.4	17	**74.1 ± 2.5**$^{(+)}$	58.3 ± 1.7	18
**Experiment 4 (long days)**						
Col-0 (wild type)	**25.3 ± 1.6**	16.9 ± 1.3	15	**29.9 ± 1.7**$^{(+)}$	16.2 ± 1.6	19
co-10	**46.6 ± 3.2**	57.0 ± 2.1	11	**50.2 ± 2.0**$^{(+)}$	50.1 ± 1.2	18
fd-3	**32.4 ± 3.0**	32.5 ± 1.5	14	**38.1 ± 1.7**$^{(+)}$	31.8 ± 1.4	20
ft-10	**42.0 ± 1.6**	46.5 ± 1.6	13	**44.2 ± 3.5**$^{(+)}$	37.2 ± 2.1	18
**Experiment 5 (long days)**						
Col-0 (wild type)	**24.7 ± 1.0**	16.9 ± 1.3	15	**27.3 ± 1.5**$^{(+)}$	16.2 ± 1.6	19
35S::MIR156	**31.0 ± 2.3**	34.0 ± 2.7	17	**38.1 ± 2.2**$^{(+)}$	39.5 ± 6.7	20
**Experiment 6 (long days)**						
Col-0 (wild type)	**22.8 ± 1.4**	18.3 ± 3.3	19			
nlp6-2	**23.8 ± 1.6**	18.2 ± 3.3	20			
nlp7-1	**27.6 ± 1.9**	21.0 ± 3.2	20			
nlp6-2;nlp7-1	**30.4 ± 2.4**	21.4 ± 4.7	19			
**Experiment 7 (short days)**						
Col-0 (wild type)	**64.1 ± 1.2**	70.7 ± 2.2	18			
nlp6-2	**70.2 ± 1.4**	73.3 ± 3.4	20			
nlp7-1	**73.4 ± 1.5**	71.3 ± 4.4	19			
nlp6-2;nlp7-1	**80.8 ± 2.0**	72.9 ± 2.2	20			

Abbreviations: DTB, days to bolting in bold as referred to in the main text; TLN, total leaf number; n, number of individuals; (+/-), presence or absence of significance based on Students t-test calculated between plants grown on the ON (optimal N soil) and LN (limited N soil), respectively (+ : p-value ≤ 0.05, - : p-value > 0.05); n.f., 100% not flowering until the end of the experiment (120 days after germination).

Table S3 Analyses of upstream intergenic regions of selected flowering time gene loci.

**gen**	**NRE**	**5′ dist. to ATG**	**trans. start**
consensus	-------------**tGaCCctT**---(**n**)----------------**AAGaG**-------------		
SPL3_1	TATTTATTTT---TGTCCATT---TTTTCTT------------AAGAT---ATTATTTCAA	1,835	1,494
SPL3_2	AAAATAGATT---TGTCCCTT---ACCTCT-------------AAGAG---ATATAGAGCA	1,522	1,181
SPL4_1	CGGATAGTTA---CGTCCGTT---TTGTTTGCTAACGTAAA--AAGAG---CGACGCGGGG	2,040	1,981
SPL5_1	TTCTTTAGCT---GGTCCAAT---CTC----------------AAGAC---TCTTTACTTG	1,210	1,117
SPL5_2	CTTGTTTAAG---TGACCAGT---AAGACTTGCGATGA-----AAGGG---ATCTGCAGTC	1,166	1,074
SPL5_3	TCGTTTGCAT---TGACCCTT---TATAGCTA-----------AAGAG---ATCTCATTGT	971	879
SPL5_4	CAACAATAGA---TGACCACT---AGGGTTGCAGG--------AAGAG---GAATCCAATA	625	533
GI_1	ATTACAATTT---AGCCCAGT---TACATAACTCCACGT----AAGGT---TTGAGCCCAT	1,731	1,177
FDP_1	ATACTAGAAA---TGACCATT---GAGAGAC------------AAGAA---GTTCACAATC	214	214
TOE2_1	GGACATGGGC---CGTCCGAT---CAGATATGTGACCT-----AAGAT---GGCTGAGAGA	2,659	2,195
TOE3_1	TGAATTTACT---TGCCCTTT---TTATATAA-----------AAGAA---AAATGTGATG	334	19
SMZ_1	GGAAAAGGGT---TGTCCTTT---GGCAAAATA----------AAGAA---AAGAAAAAGA	1,814	1,813
TEM1_1	AATTATTCTT---TGTCCTTT---AGTGAAAACATT-------AAGAA---AAATTCATCT	525	460
RGL1_1	TGGGCTATGA---AGACCATT---CTATC--------------AAGTG---CACTCACATG	2,450	2,318
GNL_1	CAATTCGCAT---GGTCCCTT---ACTTGGTTCTGTCAA----AAGAA---AGGTCTAGAG	2,427	2,427
GID1C_1	ATCCTTTGAT---TGAACCTG---TAA----------------AAGAG---AGAAGAAGCC	1,669	1,502
GA20OX1_1	AACCTCTAGT---GGTCCAAT---GGTTA--------------AAGAT---CGTTAGTTTA	2,987	2,938
GA20OX1_2	GATGGAGTAA---TGTCCTTT---TCTAAGAAAATC-------AAGAG---AAAGAAAACA	2,594	2,545
GA20OX2_1	TAGTTTTTCT---TGTCCTTT---AGATTGAGACCA-------AAGAT---TTTGTTCAAT	257	195
FRI_1	AACAAGAGAC---TGACCGAT---CATAAGAG-----------AAGAG---AGCTTCAAGG	1,180	1,180
SVP_1	TCTGACACTT---TGACCAAT---CAAAACTCA----------AAGAC---CTCACCAGTT	2,660	1,595
MIR156F_1	ATAGTAACAT---TGACCAAT---TTTCATTT-----------AAGAT---ACATTAGCCG	n.a.	3,326
MIR156F_2	ATCTTTTTTG---CGTCCATT---ATCTGC-------------AAGAT---TATCTGTATC	n.a.	114
ELF4_1	CTCGCCTCGG---TGTCCGAT---TCTACTCAG----------AAGAT---ATTTACTATA	75	43
MAF5_1	GCCCAGATTC---GGCCCATT---TAACTA-------------AAGAT---TATACCAGTT	1,151	1,005

Sequences of putative nitrate-responsive cis-elements (NREs) were identified in upstream intergenic region with a maximal distance of 3000bp to the ATG of several flowering genes and transgenic plants for synthetic promoter-reporters were generated, carrying four copies of the respective NRE fused to the 35S minimal promoter driving the *GUS* reporter gene based on a published protocol (Konishi & Yanagisawa, 2010).

Abbreviations & gene identifier: NRE (nitrate-responsive cis-element), n.a. (not applicable), *SPL3* (At2g33810), *SPL4* (At1g53160), *SPL5* (At3g15270), *GI* (At1g22770), *FDP* (At2g17770), *TOE2* (At5g60120), *TOE3* (At5g67180), *SMZ* (At3g54990), *TEM1* (At1g25560), *RGL1* (At1g66350), *GNL* (At4g26150), *GID1C* (At5g27320), *GA20Ox1* (At4g25420), *GA20Ox2* (At5g51810), *FRI* (At4g00650), *SVP* (At2g22540), *MIR156F* (At5g26147), *ELF4* (At2g40080), *MAF5* (At5g65080).

Table S4 List of genes associated with the regulation of flowering time analyzed for Table S3.

Genes presented in italic contain an nitrate responsive elements (NRE) within 3kb upstream of ATG and are part of Table S3. Gene abbreviations marked with (1) contain an NRE beyond this limit; those marked with (2) carry an NRE within the downstream intergenic region.

abbreviation	name	AGI	length of upstream intergenic region
AGL19	AGAMOUS LIKE 19	At4g22950	2598
AGL24	AGAMOUS LIKE 24	At4g24540	2088
AP1	APETALA 1	At1g69120	3627
AP2	APETALA 2	At4g36920	5597
CAL	CAULIFLOWER	At1g26310	1697
CCA1	CIRCADIAN CLOCK ASSOCIATED 1	At2g46830	863
CCR1	CAROTENOID CHLOROPLAST REGULATORY 1	At1g77300	2694
CDF1[(]	CYCLING DOF FACTOR 1	At5g62430	1679
CLF	CURLY LEAF	At2g23380	478
CO	CONSTANS	At5g15840	487
COP1[(2)]	CONSTITUTIVE PHOTOMORPHOGENIC 1	At2g32950	523
COP2	CONSTITUTIVE PHOTOMORPHOGENIC 2	At3g54720	1443
CRY1	CRYPTOCHROME 1	At4g08920	5559
CRY2	CRYPTOCHROME 2	At2g04400	1176
DDF1	DWARF AND DELAYED IN FLOWERING 1	At1g12610	3759
EBS	EARLY BOLTING IN SHORT DAYS	At4g22140	907
ELF4	*EARLY FLOWERING 4*	*At2g40080*	*1580*
ELF7	EARLY FLOWERING 7	At1g79730	397
ELF9	EARLY FLOWERING 9	At5g16260	547
EMF1	EMBRYONIC FLOWER 1	At5g11530	6739
EMF2	EMBRYONIC FLOWER 2	At5g51230	886
ESD4	EARLY IN SHORT DAYS	At4g15880	1821
FCA	FLOWERING CONTROL LOCUS A	At4g16280	1267
FD	ATBZIP14	At4g35900	3912
FDP	*FD PARALOG*	*At2g17770*	*5402*
FKF1	FLAVIN-BINDING KELCH REPEAT F BOX 1	At1g68050	3504
FLC	FLOWERING LOCUS C	At5g10140	2643
FLD	FLOWERING LOCUS D	At3g10390	359
FLK	FLOWERING LOCUS KH DOMAIN	At3g04610	1253
FLM	FLOWERING LOCUS M	At1g77080	2169
FPF1	FLOWERING PROMOTING FACTOR 1	At5g24860	3714
FRI[(2)]	*FRIGIDA*	*At4g00650*	*1236*
FT[(2)]	FLOWERING LOCUS T	At1g65480	5509
FUL[(1)]	FRUITFUL	At5g60910	5182
FWA	FLOWERING WAGENINGEN	At4g25530	3098
GA1	GA REQUIRING 1	At4g02780	2649
GA20OX1	*GIBBERELLIN 20-OXIDASE 1*	*At4g25420*	*5951*
GA20OX2	*GIBBERELLIN 20-OXIDASE 2*	*At5g51810*	*6621*
GAI[(1)]	GIBBERELLIC ACID INSENSITIVE	At1g14920	5613
GI	*GIGANTEA*	*At1g22770*	*3381*
GID1A	GA INSENSITIVE DWARF 1A	At3g05120	2962
GID1B	GA INSENSITIVE DWARF 1B	At3g63010	3890
GID1C[(2)]	*GA INSENSITIVE DWARF 1C*	*At5g27320*	*2692*
GNC[(1)]	GATA NITRATE-INDUCIBLE CARBON METABOLISM-INVOLVED	At5g56860	6610
GNL[(1)]	*GNC-LIKE*	*At4g26150*	*5283*
LD	LUMINIDEPENDENS	At4g02560	369
LFY	LEAFY	At5g61850	2354
LHY	LATE ELONGATED HYPOCOTYL	At1g01060	1690
MAF5	*MADS AFFECTING FLOWERING 5*	*At5g65080*	*1475*
MFT	MOTHER OF FT AND TFL1	At1g18100	5784
MIR156A[(2)]	MICRO RNA 156A	At2g25095	2970
MIR156B	MICRO RNA 156B	At4g30972	356
MIR156C[(2)]	MICRO RNA 156C	At4g31877	3530
MIR156D	MICRO RNA 156D	At5g10945	2540
MIR156E	MICRO RNA 156E	At5g11977	3457
MIR156F	*MICRO RNA 156F*	*At5g26147*	*4489*
MIR156G	*MICRO RNA 156G*	At2g19425	-
MIR156H	MICRO RNA 156H	At5g55835	-
MIR172A	MICRO RNA 172A	At2g28056	6381
MIR172B[(2)]	MICRO RNA 172B	At5g04275	3445
PHYA	PHYTOCHROME A	At1g09570	2454
PHYB	PHYTOCHROME B	At2g18790	1960
PIE1	PHOTOPERIOD-INDEPENDENT EARLY FLOWERING 1	At3g12810	813
RGA	REPRESSOR OF GA	At2g01570	7456
RGL1	*RGA LIKE 1*	*At1g66350*	*5626*
RGL2	RGA LIKE 2	At3g03450	1161
RGL3	RGA LIKE 3	At5g17490	1491
SE	SERRATE	At2g27100	730
SEF	SERRATED LEAVES AND EARLY FLOWERING	At5g37055	353
SEP2	SEPALATA 2	At3g02310	3246
SHL1	SHORT LIFE	At4g39100	454
SMZ	*SCHLAFMÜTZE*	*At3g54990*	*4985*
SNZ	SCHNARCHZAPFEN	At2g39250	1115
SOC1[(1,2)]	SUPRESSOR OF OVEREXPRESSION OF CONSTANS1	At2g45660	4515
SPA1	SUPPRESSOR OF PHYA 1	At2g46340	1666
SPA3	SUPPRESSOR OF PHYA 3	At3g15354	1112
SPA4	SUPPRESSOR OF PHYA 4	At1g53090	1528
SPL3	*SQUAMOSA PROMOTER BINDING LIKE PROTEIN3*	*At2g33810*	*2911*
SPL4	*SQUAMOSA PROMOTER BINDING LIKE PROTEIN4*	*At1g53160*	*3879*
SPL5	*SQUAMOSA PROMOTER BINDING LIKE PROTEIN5*	*At3g15270*	*2711*
SPL9	SQUAMOSA PROMOTER BINDING LIKE PROTEIN9	At2g42200	3406
SPL10	SQUAMOSA PROMOTER BINDING LIKE PROTEIN10	At1g27370	1905
SPL15	SQUAMOSA PROMOTER BINDING LIKE PROTEIN15	At3g57920	1397
SPY	SPINDLY	At3g11540	2358
SUF4	SUPPRESOR OF FRI 4	At1g30970	1077
SVP	*SHORT VEGETATIVE PHASE*	*At2g22540*	*2972*
TEM1	*TEMPRANILLO 1*	*At1g25560*	*8224*
TEM2[(1)]	TEMPRANILLO 2	At1g68840	4891
TFL1	TERMINAL FLOWER1	At5g03840	2543
TFL2	TERMINAL FLOWER 2	At5g17690	1043
TOC1	TIMING OF CAB EXPRESSION 1	At5g61380	1882

Table S4 List continued

abbreviation	name	AGI	length of upstream intergenic region
TOE1	TARGET OF EARLY ACTIATION TAGGED 1	At2g28550	7149
TOE2[(2)]	*TARGET OF EARLY ACTIATION TAGGED 2*	*At5g60120*	*5215*
TOE3	*TARGET OF EAT3*	*At5g67180*	*5200*
TPS1[(1)]	TREHALOSE PHOSPHATE SYNTHASE1	At1g78580	6766
TSF	TWIN SISTER OF FT	At4g20370	1551
UFO	UNUSUAL FLORAL ORGANS	At1g30950	5479
VIN3	VERNALIZATION INSENSITIVE 3	At5g57380	4169
VRN1	REDUCED VERNALIZATION RESPONSE 1	At3g18990	1960
VRN2	REDUCED VERNALIZATION RESPONSE 2	At4g16845	1184

REFERENCES

Konishi M, Yanagisawa S. 2010. Identification of a nitrate-responsive cis-element in the Arabidopsis NIR1 promoter defines the presence of multiple cis-regulatory elements for nitrogen response. *Plant J* **63**(2): 269-282.

Mutasa-Gottgens E, Hedden P. 2009. Gibberellin as a factor in floral regulatory networks. *J Exp Bot* **60**(7): 1979-1989.

Rowan DD, Cao M, Lin-Wang K, Cooney JM, Jensen DJ, Austin PT, Hunt MB, Norling C, Hellens RP, Schaffer RJ, et al. 2009. Environmental regulation of leaf colour in red 35S:PAP1 Arabidopsis thaliana. *New Phytol* **182**(1): 102-115.
